# Supplementary material for: Family-based genome-wide association study designs for increased power and robustness
Source: Nat Genet. 2025 Mar 10;57(4):1044–52. doi: 10.1038/s41588-025-02118-0 (PMC11985344; doi:10.1038/s41588-025-02118-0)
Supplement: Supplementary file 1 — Supplementary Tables and Notes. [file 41588_2025_2118_MOESM1_ESM.pdf]

nature genetics

Supplementary information

https://doi.org/10.1038/s41588-025-02118-0

Family-based genome-wide association study designs for increased power and robustness

In the format provided by the authors and unedited

Contents

Supplementary Tables 2

Supplementary Note 6

1 Analysis of simulations based on UK Biobank 'White British' sample 6

2 Estimators 6
2.1 Theoretical effective sample size for the unified estimator 6
2.1.1 Imputation from sibling pairs 7
2.1.2 Imputation from parent-offspring pairs 8
2.2 Modeling average NTC with imputation from parent-offspring pairs in the Young et al. estimator 10
2.3 The non-transmitted estimator 11
2.3.1 Effect of population structure 11
2.3.2 Properties of the non-transmitted estimator 12
2.3.3 Effect of admixture 14
2.4 The robust estimator 16
2.4.1 Implementation of the robust estimator 17
2.5 The sib-difference estimator 18
2.5.1 Equivalence of the sib-difference estimator to the robust estimator in sibling pair samples 18

3 Linear imputation using distant relationships 19

4 Educational Achievement outcome in MCS 20

Supplementary Tables

Table S1: Results from analysis of UK Biobank data.

Phenotype	Sib. corr.	$\frac{n_{\text{eff;unified}}}{n_{\text{eff;sib-difference}}}$	$\frac{n_{\text{eff;Young2022}}}{n_{\text{eff;sib-difference}}}$	$\frac{n_{\text{eff;unified}}}{n_{\text{eff;Young2022}}}$	$\frac{n_{\text{eff;nontransmitted}}}{n_{\text{eff;sib-difference}}}$	$\frac{n_{\text{eff;robust}}}{n_{\text{eff;sib-difference}}}$
Subjective well-being	0.0921	2.0653	1.4526	1.4217	1.2740	1.2087
Number of children (male)	0.1297	1.9965	1.4003	1.4257	1.2575	1.1917
Neuroticism	0.1376	1.9648	1.4106	1.3929	1.2515	1.2081
Self-rated health	0.1492	1.9085	1.3810	1.3820	1.2318	1.1917
Number of children (female)	0.1505	1.9519	1.3929	1.4013	1.2580	1.2103
Diastolic blood pressure	0.1580	1.8942	1.3788	1.3738	1.2309	1.1922
Systolic blood pressure	0.1587	1.8887	1.3711	1.3775	1.2234	1.1846
Drinks-per-week	0.1692	2.0392	1.4328	1.4232	1.2577	1.1917
Non-HDL cholesterol	0.1764	1.8819	1.3702	1.3734	1.2246	1.1846
Cigarettes-per-day	0.1830	1.9694	1.3952	1.4115	1.2343	1.1600
Ever-smoked	0.1838	1.8363	1.3549	1.3554	1.2166	1.1820
Myopia	0.1960	1.8967	1.3706	1.3838	1.2219	1.1839
Household Income	0.2411	1.8655	1.3616	1.3701	1.2218	1.1924
BMI	0.2782	1.7389	1.3101	1.3273	1.1876	1.1653
HDL cholesterol	0.2807	1.7640	1.3108	1.3458	1.1860	1.1583
Age-at-first-birth (women)	0.2989	1.8505	1.3405	1.3805	1.2222	1.1627
Cognitive ability	0.3093	1.8359	1.3463	1.3637	1.2092	1.1703
Educational attainment (years)	0.4023	1.6092	1.2240	1.3147	1.1287	1.1161
Height	0.4978	1.4696	1.1804	1.2450	1.1075	1.1033

Note. Phenotypic correlations of siblings ('sib. corr.') and effective sample size ratios between different estimators. $\frac{n_{\text{eff;unified}}}{n_{\text{eff;sib-difference}}}$, $\frac{n_{\text{eff;Young2022}}}{n_{\text{eff;sib-difference}}}$ and $\frac{n_{\text{eff;unified}}}{n_{\text{eff;Young2022}}}$ were obtained from summary statistics derived from UKB 'white British' subsample, whereas $\frac{n_{\text{eff;nontransmitted}}}{n_{\text{eff;sib-difference}}}$ and $\frac{n_{\text{eff;robust}}}{n_{\text{eff;sib-difference}}}$ were obtained from UKB sample without ancestry restriction. Abbreviations: HDL, high density lipoprotein; BMI, body mass index.

Table S2: Computational runtime of the four methods implemented in `snipar`.

Method	Sample size	Density	REML runtime (s)	Effect estimation runtime (s)
Sib-difference	41,209	5.233×10^{-5}	22.09	186.13
Robust	45,940	4.536×10^{-5}	23.76	544.85
Young 2022	39,525	5.294×10^{-5}	23.68	111.90
Unified	406,878	3.840×10^{-6}	173.93	844.91

Note. Runtimes for REML variance component estimation and SNP effect estimation (parallelized over 4 CPUs) are separately recorded for the four methods applied to UK Biobank height measurement and genotyping array data on chromosome 22 (10,911 SNPs). Sample size refers to the number of individuals with non-missing height measurements, whereas density refers to the number of nonzero entries relative to the total number of entries in the sparse GRM.

Table S3: Genetic correlations among direct effect estimates from different estimators for height and EA in the UK Biobank data.

Methods	Height		EA	
	r_g	SE	r_g	SE
Young 2022 & unified	0.9990	0.0045	1.0329	0.0229
Young 2022 & sib-difference	1.0057	0.0064	1.0214	0.0366
Young 2022 & robust	1.0034	0.0053	0.9925	0.0265
Unified & sib-difference	0.9917	0.0084	1.0025	0.0534
Unified & robust	0.9816	0.0079	0.9331	0.0419
Sib-difference & robust	1.0145	0.0029	1.0672	0.0235

Note. Genetic correlations r_g (with standard errors SE) among direct effect estimates obtained from the Young et al. estimator, the unified estimator, the sib-difference estimator, and the robust estimator are computed using LDSC with the default settings. We used the LD reference panel computed from phased haplotypes for the UK Biobank genotyping array SNPs.

Table S4: MCS PGI analysis results.

Standardized population effects

Phenotype	Sib-difference EUR $\hat{\beta}_{PGI}$	S.E.	p-value	Sib-difference SAS $\hat{\beta}_{PGI}$	S.E.	p-value	Sib-difference Diff. $\hat{\beta}_{PGI}$	S.E.	p-value
BMI	0.0955	0.0138	0.0000	0.1093	0.0383	0.0043	-0.0138	0.0407	0.7342
Height	0.2395	0.0135	0.0000	0.3054	0.0367	0.0000	-0.0659	0.0391	0.0918
EA	0.0374	0.0156	0.0167	0.0408	0.0405	0.3140	-0.0034	0.0434	0.9375

Phenotype	Robust EUR $\hat{\beta}_{PGI}$	S.E.	p-value	Robust SAS $\hat{\beta}_{PGI}$	S.E.	p-value	Robust Diff. $\hat{\beta}_{PGI}$	S.E.	p-value
BMI	0.0973	0.0138	0.0000	0.1166	0.0383	0.0023	-0.0193	0.0407	0.6355
Height	0.2524	0.0134	0.0000	0.3211	0.0365	0.0000	-0.0687	0.0389	0.0770
EA	0.0461	0.0156	0.0031	0.0109	0.0406	0.7875	0.0352	0.0435	0.4178

Phenotype	Young et al. 2022 EUR $\hat{\beta}_{PGI}$	S.E.	p-value	Young et al. 2022 SAS $\hat{\beta}_{PGI}$	S.E.	p-value	Young et al. 2022 Diff. $\hat{\beta}_{PGI}$	S.E.	p-value
BMI	0.1028	0.0138	0.0000	0.1269	0.0382	0.0009	-0.0241	0.0406	0.5533
Height	0.2521	0.0134	0.0000	0.3003	0.0367	0.0000	-0.0482	0.0391	0.2180
EA	0.0371	0.0156	0.0175	0.0186	0.0405	0.6465	0.0185	0.0435	0.6701

Phenotype	Unified EUR $\hat{\beta}_{PGI}$	S.E.	p-value	Unified SAS $\hat{\beta}_{PGI}$	S.E.	p-value	Unified Diff. $\hat{\beta}_{PGI}$	S.E.	p-value
BMI	0.1253	0.0138	0.0000	0.1415	0.0381	0.0002	-0.0163	0.0405	0.6882
Height	0.2524	0.0134	0.0000	0.3589	0.0360	0.0000	-0.1065	0.0384	0.0055
EA	0.0504	0.0156	0.0012	0.0263	0.0405	0.5166	0.0241	0.0434	0.5789

Phenotype	Standard GWAS EUR $\hat{\beta}_{PGI}$	S.E.	p-value	Standard GWAS SAS $\hat{\beta}_{PGI}$	S.E.	p-value	Standard GWAS Diff. $\hat{\beta}_{PGI}$	S.E.	p-value
BMI	0.2356	0.0135	0.0000	0.2340	0.0374	0.0000	0.0016	0.0398	0.9672
Height	0.4125	0.0126	0.0000	0.4188	0.0350	0.0000	-0.0063	0.0372	0.8662
EA	0.2279	0.0152	0.0000	0.0803	0.0404	0.0470	0.1476	0.0432	0.0006

Standardized direct effects

Phenotype	Sib-difference EUR $\hat{\delta}_{PGI}$	S.E.	p-value	Robust EUR $\hat{\delta}_{PGI}$	S.E.	p-value	Young et al. 2022 EUR $\hat{\delta}_{PGI}$	S.E.	p-value	Unified EUR $\hat{\delta}_{PGI}$	S.E.	p-value	Standard GWAS EUR $\hat{\delta}_{PGI}$	S.E.	p-value
BMI	0.1092	0.0218	0.0000	0.0926	0.0220	0.0000	0.1039	0.0218	0.0000	0.1415	0.0219	0.0000	0.2254	0.0216	0.0000
Height	0.2316	0.0220	0.0000	0.2289	0.0217	0.0000	0.2279	0.0219	0.0000	0.2358	0.0216	0.0000	0.3662	0.0207	0.0000
EA	0.0567	0.0253	0.0251	0.0576	0.0253	0.0227	0.0494	0.0250	0.0486	0.0476	0.0252	0.0589	0.0789	0.0244	0.0012

Standardized population effects (with PC adjustment)

Phenotype	Sib-difference EUR $\hat{\beta}_{PGI}$	S.E.	p-value	Sib-difference SAS $\hat{\beta}_{PGI}$	S.E.	p-value	Sib-difference Diff. $\hat{\beta}_{PGI}$	S.E.	p-value
BMI	0.0948	0.0139	0.0000	0.0914	0.0399	0.0219	0.0034	0.0422	0.9366
Height	0.2405	0.0135	0.0000	0.2852	0.0373	0.0000	-0.0447	0.0397	0.2604
EA	0.0342	0.0157	0.0290	0.0359	0.0406	0.3760	-0.0017	0.0435	0.9683

Phenotype	Robust EUR $\hat{\beta}_{PGI}$	S.E.	p-value	Robust SAS $\hat{\beta}_{PGI}$	S.E.	p-value	Robust Diff. $\hat{\beta}_{PGI}$	S.E.	p-value
BMI	0.0962	0.0139	0.0000	0.0998	0.0397	0.0120	-0.0036	0.0420	0.9326
Height	0.2533	0.0135	0.0000	0.2989	0.0371	0.0000	-0.0456	0.0395	0.2486
EA	0.0432	0.0157	0.0058	-0.0064	0.0409	0.8765	0.0495	0.0438	0.2578

Phenotype	Young et al. 2022 EUR $\hat{\beta}_{PGI}$	S.E.	p-value	Young et al. 2022 SAS $\hat{\beta}_{PGI}$	S.E.	p-value	Young et al. 2022 Diff. $\hat{\beta}_{PGI}$	S.E.	p-value
BMI	0.1021	0.0139	0.0000	0.1174	0.0397	0.0031	-0.0153	0.0421	0.7169
Height	0.2522	0.0135	0.0000	0.2867	0.0370	0.0000	-0.0345	0.0393	0.3810
EA	0.0347	0.0156	0.0263	0.0079	0.0405	0.8446	0.0268	0.0434	0.5368

Phenotype	Unified EUR $\hat{\beta}_{PGI}$	S.E.	p-value	Unified SAS $\hat{\beta}_{PGI}$	S.E.	p-value	Unified Diff. $\hat{\beta}_{PGI}$	S.E.	p-value
BMI	0.1232	0.0138	0.0000	0.1363	0.0393	0.0005	-0.0130	0.0417	0.7543
Height	0.2520	0.0135	0.0000	0.3369	0.0369	0.0000	-0.0849	0.0392	0.0305
EA	0.0484	0.0156	0.0020	0.0122	0.0403	0.7614	0.0361	0.0432	0.4036

Phenotype	Standard GWAS EUR $\hat{\beta}_{PGI}$	S.E.	p-value	Standard GWAS SAS $\hat{\beta}_{PGI}$	S.E.	p-value	Standard GWAS Diff. $\hat{\beta}_{PGI}$	S.E.	p-value
BMI	0.2353	0.0136	0.0000	0.2148	0.0388	0.0000	0.0205	0.0411	0.6180
Height	0.4113	0.0127	0.0000	0.3994	0.0362	0.0000	0.0119	0.0383	0.7559
EA	0.2306	0.0153	0.0000	0.0596	0.0405	0.1411	0.1710	0.0433	0.0001

Standardized direct effects (with PC adjustment)

Phenotype	Sib-difference EUR $\hat{\delta}_{PGI}$	S.E.	p-value	Robust EUR $\hat{\delta}_{PGI}$	S.E.	p-value	Young et al. 2022 EUR $\hat{\delta}_{PGI}$	S.E.	p-value	Unified EUR $\hat{\delta}_{PGI}$	S.E.	p-value	Standard GWAS EUR $\hat{\delta}_{PGI}$	S.E.	p-value
BMI	0.1091	0.0219	0.0000	0.0912	0.0220	0.0000	0.1026	0.0218	0.0000	0.1410	0.0220	0.0000	0.2254	0.0217	0.0000
Height	0.2358	0.0219	0.0000	0.2345	0.0216	0.0000	0.2323	0.0219	0.0000	0.2401	0.0216	0.0000	0.3662	0.0207	0.0000
EA	0.0548	0.0253	0.0306	0.0562	0.0253	0.0261	0.0490	0.0251	0.0504	0.0487	0.0252	0.0530	0.0772	0.0244	0.0015

Note. We computed the standardized population effect PGI estimates $\hat{\beta}_{PGI}$'s, standard errors S.E.'s and p-values for BMI, height and EA in both the European and South Asian samples in MCS. We also calculated the differences (diff.) of the estimates from the two subsamples and the corresponding S.E.'s and p-values in a two-sided Z-test. We only computed the direct effect PGI estimates from the European sample due to a lack of first-degree relationships in the South Asian sample. In the bottom two tables, 20 principal components were included in the PGI regression model.

Supplementary Note

1 Analysis of simulations based on UK Biobank 'White British' sample

We examine the proposed unified estimator on a number of populations derived from the UK Biobank 'white British' subsample. The datasets are simulated under different assumptions, including: random mating, assortative mating, population stratification, etc. Phenotypes are generated from 1,500 SNPs with randomly drawn direct and parental indirect genetic effects under different correlation assumptions. The exact procedure can be found in [1]. The final output is 49,991 sibling pairs without observed parental genotypes.

To investigate the performance of the unified estimator, we randomly remove one sibling's genotype from each sibling pair for half of the pairs, leaving us with $n_0 = 24,996$ genotyped sibling pairs and $n_1 = 24,995$ genotyped singletons. We then impute the parental genotypes of the sibling pairs with unphased IBD data using the methods described in Young et al.[1], and and linearly impute those of singletons (Methods). The results are given in Table SN1. It can be seen that the observed relative effective sample size is negatively correlated with sibling correlation, with the 5th scenario observing the lowest sample size gain and the highest sibling correlation. This is consistent with the trends in Figure 2 in the main text.

We compared the estimated direct SNP effects to the true causal effects. We regress the estimates onto the true values, inverse-weighted by the estimation variance. The regression coefficients and standard errors are given in the last two columns of Table SN1. For all simulation scenarios, the regression coefficient is close to 1, and within two standard errors of 1, indicating little to no inflation or deflation of direct effect estimates under these scenarios.

2 Estimators

2.1 Theoretical effective sample size for the unified estimator

In the main text we claimed that performing family-based GWAS on the combined sample yields the same results as meta-analyzing results from family-based GWAS on the related sample and standard univariate GWAS on singletons. To see that, we first look at a general case where we are given two sets of effect estimates $\mathbf{z}_0$ and $\mathbf{z}_1$ obtained from independent datasets, with

$$\mathbf{z}_0 \sim \mathcal{N}(\mathbf{A}_0\theta, \boldsymbol{\Sigma}_0) \quad \text{and} \quad \mathbf{z}_1 \sim \mathcal{N}(\mathbf{A}_1\theta, \boldsymbol{\Sigma}_1),$$

where $\mathbf{A}_i$'s are some linear transformations and $\boldsymbol{\Sigma}_i$'s are estimation covariance matrices. By [1], the MLE of the true effect sizes θ is given by

$$\hat{\theta} = \left(\sum_{i=0}^{1} \mathbf{A}_i^\top \boldsymbol{\Sigma}_i^{-1} \mathbf{A}_i\right)^{-1} \left(\sum_{i=0}^{1} \mathbf{A}_i^\top \boldsymbol{\Sigma}_i^{-1} \mathbf{z}_i\right), \quad \mathrm{Var}(\hat{\theta}) = \left(\sum_{i=0}^{1} \mathbf{A}_i^\top \boldsymbol{\Sigma}_i^{-1} \mathbf{A}_i\right)^{-1}, \tag{1}$$

Table SN1: Results from simulated datasets.

No.	Scenario parameters	Sib. corr.	$\frac{n_{\text{eff}}(\text{sibs+singletons})}{n_{\text{eff}}(\text{sibs})}$	Coefficient ($\hat{\delta} \sim \delta$)	S.E.
1	h^2=0.75	0.389	1.074	0.995	0.009
2	h^2=0.5; VT=0.25	0.512	1.060	0.997	0.012
3	h^2=0.5; AM with r_y=0.5	0.386	1.077	0.993	0.011
4	h^2=0.5; pop. strat.=0.3	0.549	1.052	0.999	0.009
5	h^2=0.75; $r_{\delta,\eta}$=1	0.674	1.038	1.028	0.015

Note. Results for simulated phenotypes based on UK Biobank 'White British' sample. We imputed parental genotypes from un-phased data and IBD information, as in Young et al.[1], for $n_0 = 24,996$ independent sibling pairs, and we linearly imputed parental genotypes for $n_1 = 24,995$ singletons. Scenario 1 is simulated under random-mating with a heritability of 75%; scenario 2 is generated by vertical transmission taken to equilibrium [2]: the offspring's phenotype is affected by the parent's phenotype with a coefficient of 0.25 and a heritability in the first generation of 50%; scenario 3 is generated by assortative mating taken to equilibrium with a phenotypic correlation between parents of $r_y = 0.5$; scenario 4 is simulated under population stratification with 30% of the phenotypic variance explained by environmental difference between regions; in scenario 5, direct effects and IGEs from parents are perfectly correlated, $r_{\delta,\eta}$=1, and together explain 75% of the phenotypic variance. For more details please see the supplementary note in [1]. The phenotypic correlation between siblings is given in the 'Sib. corr.' column. The relative effective sample size when using the combined sample of sibling pairs and singletons compared to using the sibling pairs alone (with imputation) is given in the $\frac{n_{\text{eff}}(\text{sibs+singletons})}{n_{\text{eff}}(\text{sibs})}$ column, averaged over all SNPs with varying allele frequencies. The coefficient from regression of direct effect estimates (using the combined sample of sibling pairs and singletons) is given by the 'Coefficient ($\hat{\delta} \sim \delta$)' column, and its standard error is given in the 'S.E.' column.

if $\sum_{i=0}^{1} \mathbf{A}_i^\top \mathbf{\Sigma}_i^{-1}\mathbf{A}_i$ is invertible. Now suppose we have $\mathbf{X}_0' = \mathbf{X}_0\mathbf{A}_0$ and $\mathbf{X}_1' = \mathbf{X}_1\mathbf{A}_1$, which are transformed from raw design matrices $\mathbf{X}_0$ and $\mathbf{X}_1$. Combining them into one dataset, we get

$$\mathbf{y} = \begin{bmatrix}\mathbf{y}_0\\ \mathbf{y}_1\end{bmatrix}, \mathbf{X}' = \begin{bmatrix}\mathbf{X}_0'\\ \mathbf{X}_1'\end{bmatrix}.$$

Then we have

$$\mathbf{y} \sim \mathcal{N}\left(\mathbf{X}'\theta, \mathbf{V} = \begin{bmatrix}\mathbf{V}_0 & \mathbf{0}\\ \mathbf{0} & \mathbf{V}_1\end{bmatrix}\right).$$

Thus,

$$(\mathbf{X}'^\top\mathbf{V}^{-1}\mathbf{X}')^{-1} = (\mathbf{X}_0'\mathbf{V}_0^{-1}\mathbf{X}_0' + \mathbf{X}_1'^\top\mathbf{V}_1^{-1}\mathbf{X}_1')^{-1} \tag{2}$$

and

$$\mathbf{X}'^\top\mathbf{V}^{-1}\mathbf{y} = \mathbf{X}_0'\mathbf{V}_0^{-1}\mathbf{y}_0 + \mathbf{X}_1'^\top\mathbf{V}_1^{-1}\mathbf{y}_1. \tag{3}$$

Suppose we want to estimate the direct effect and the average non-transmitted coefficient. So for the related sample, we have $\mathbf{A}_0 = \mathbf{I}$, since we have enough information to estimate both parameters. For singletons, since the imputed parental genotype is a linear function of the proband's genotype, the estimated effect will be the sum of the two effects, meaning that $\mathbf{A}_1 = \begin{bmatrix}1 & 1\end{bmatrix}$. Also, assume that we only model the sibling variance component and the residual variance component. This gives $\mathbf{V}_0 = \sigma_s^2\mathbf{Z}_0\mathbf{Z}_0^\top + \sigma_\epsilon^2\mathbf{I}$, $\mathbf{V}_1 = \sigma_\epsilon^2\mathbf{I}$, $\mathbf{\Sigma}_0^{-1} = \mathbf{X}_0^\top\mathbf{V}_0^{-1}\mathbf{X}_0$ and $\mathbf{\Sigma}_1^{-1} = \mathbf{X}_1^\top\mathbf{V}_1^{-1}\mathbf{X}_1$, since there is no sibling pair in the sample of singletons. Combining the above, we have

$$\begin{aligned}
\mathbf{A}_0^\top\mathbf{\Sigma}_0^{-1}\mathbf{A}_0 + \mathbf{A}_1^\top\mathbf{\Sigma}_1^{-1}\mathbf{A}_1 &= \mathbf{I}\mathbf{X}_0^\top\mathbf{V}_0^{-1}\mathbf{X}_0\mathbf{I} + \begin{bmatrix}1 & 1\end{bmatrix}^\top \mathbf{X}_1^\top\mathbf{V}_1^{-1}\mathbf{X}_1\begin{bmatrix}1 & 1\end{bmatrix}\\
&= \mathbf{X}_0^\top\mathbf{V}_0^{-1}\mathbf{X}_0 + \begin{bmatrix}1 & 1\end{bmatrix}^\top \mathbf{X}_1^\top\mathbf{V}_1^{-1}\mathbf{X}_1\begin{bmatrix}1 & 1\end{bmatrix}\\
\mathbf{X}_0'\mathbf{V}_0^{-1}\mathbf{X}_0' + \mathbf{X}_1'^\top\mathbf{V}_1^{-1}\mathbf{X}_1' &= \mathbf{A}_0^\top\mathbf{X}_0^\top\mathbf{V}_0^{-1}\mathbf{X}_0\mathbf{A}_0 + \mathbf{A}_1\top\mathbf{X}_1^\top\mathbf{V}_1^{-1}\mathbf{X}_1\mathbf{A}_1\\
&= \mathbf{X}_0^\top\mathbf{V}_0^{-1}\mathbf{X}_0 + \begin{bmatrix}1 & 1\end{bmatrix}^\top \mathbf{X}_1^\top\mathbf{V}_1^{-1}\mathbf{X}_1\begin{bmatrix}1 & 1\end{bmatrix}
\end{aligned}$$

and

$$\begin{aligned}
\mathbf{A}_0^\top\mathbf{\Sigma}_0^{-1}\mathbf{y}_0 + \mathbf{A}_1^\top\mathbf{\Sigma}_1^{-1}\mathbf{y}_1 &= \mathbf{I}\mathbf{X}_0^\top\mathbf{V}_0^{-1}\mathbf{y}_0 + \begin{bmatrix}1 & 1\end{bmatrix}^\top \mathbf{X}_1^\top\mathbf{V}_1^{-1}\mathbf{y}_1\\
&= \mathbf{X}_0^\top\mathbf{V}_0^{-1}\mathbf{y}_0 + \begin{bmatrix}1 & 1\end{bmatrix}^\top \mathbf{X}_1^\top\mathbf{V}_1^{-1}\mathbf{y}_1\\
\mathbf{X}_0'\mathbf{V}_0^{-1}\mathbf{y}_0 + \mathbf{X}_1'^\top\mathbf{V}_1^{-1}\mathbf{y}_1 &= \mathbf{A}_0^\top\mathbf{X}_0^\top\mathbf{V}_0^{-1}\mathbf{y}_0 + \mathbf{A}_1\top\mathbf{X}_1^\top\mathbf{V}_1^{-1}\mathbf{y}_1\\
&= \mathbf{X}_0^\top\mathbf{V}_0^{-1}\mathbf{y}_0 + \begin{bmatrix}1 & 1\end{bmatrix}^\top \mathbf{X}_1^\top\mathbf{V}_1^{-1}\mathbf{y}_1,
\end{aligned}$$

establishing the equivalence between (1) and (2), (3). In particular, the effect estimation variance-covariance matrices from the two approaches are the same. Note that the relative effective sample size for an effect estimate is given by the ratio of the estimation variance from the singletons to that from the augmented sample. Thus, it suffices to consider sample size gain in the meta-analysis approach.

2.1.1 Imputation from sibling pairs

We give the theoretical effective sample size gain for direct effect estimate $\hat{\delta}$ from adding n_1 singletons to a sample of n_0 sibling pairs. We consider Model 2 in the main text with the parameter vector $\begin{bmatrix}\delta & \alpha\end{bmatrix}^\top$. From standard univariate GWAS on the n_1 singletons, we have

$$\hat{\theta}_1 \sim \mathcal{N}\left(\begin{bmatrix}1 & 1\end{bmatrix}\theta, \frac{\sigma_\epsilon^2}{n_1 2f(1-f)}\right). \tag{4}$$

Let r be the sibling phenotypic correlation and v be the proportion of parental genotypic variance attributable to the imputation. Then, under random-mating, family-based GWAS yields[1]:

$$\hat{\theta}_0 \sim \mathcal{N}\left(\theta, \frac{\sigma_\epsilon^2(1+r)}{8[(2-r)v+r-1]n_0 f(1-f)}\begin{bmatrix}4v(1-r) & -2(1-r)\\ -2(1-r) & 2-r\end{bmatrix}\right).$$

Following from (1), we have

$$\mathrm{Var}(\hat{\theta}) = \left\{\frac{8[(2-r)v+r-1]n_0 f(1-f)}{\sigma_\epsilon^2(1+r)}\begin{bmatrix}4v(1-r) & -2(1-r)\\ -2(1-r) & 2-r\end{bmatrix}^{-1} + \frac{2n_1 f(1-f)}{\sigma_\epsilon^2}\begin{bmatrix}1 & 1\\ 1 & 1\end{bmatrix}\right\}^{-1}.$$

If imputation is done using phased IBD data, $v = 3/4$; if unphased data is used, $v = 3/4 - f(1-f)/8$. Consider the special case where there is zero sibling phenotypic correlation, and that phased IBD data is used. Then

$$\hat{\theta}_0 \sim \mathcal{N}\left(\theta, \frac{\sigma_\epsilon^2}{n_0 f(1-f)} \begin{bmatrix} 4 & 4 \\ 4 & 6 \end{bmatrix}^{-1}\right)$$

with

$$\mathrm{Var}(\hat{\delta}_0) = \frac{3\sigma_\epsilon^2}{4n_0 f(1-f)},$$

and

$$\begin{aligned}\mathrm{Var}(\hat{\theta}) &= \left\{\frac{n_0 f(1-f)}{\sigma_\epsilon^2} \begin{bmatrix} 4 & 4 \\ 4 & 6 \end{bmatrix} + \frac{2n_1 f(1-f)}{\sigma_\epsilon^2} \begin{bmatrix} 1 & 1 \\ 1 & 1 \end{bmatrix}\right\}^{-1} \\ &= \frac{\sigma_\epsilon^2}{f(1-f)} \begin{bmatrix} 4n_0 + 2n_1 & 4n_0 + 2n_1 \\ 4n_0 + 2n_1 & 6n_0 + 2n_1 \end{bmatrix}^{-1} \\ &= \frac{\sigma_\epsilon^2}{f(1-f)} \begin{bmatrix} \frac{3n_0+n_1}{2n_0n_1+4n_0^2} & -\frac{1}{2n_0} \\ -\frac{1}{2n_0} & \frac{1}{2n_0} \end{bmatrix}\end{aligned}$$

with

$$\mathrm{Var}(\hat{\delta}) = \frac{\sigma_\epsilon^2}{n_0 f(1-f)} \frac{3n_0 + n_1}{2n_0 n_1 + 4n_0^2}.$$

Therefore, the relative effective sample size is given by

$$\frac{\mathrm{Var}(\hat{\delta}_0)}{\mathrm{Var}(\hat{\delta})} = \frac{\frac{3\sigma_\epsilon^2}{4n_0 f(1-f)}}{\frac{\sigma_\epsilon^2}{f(1-f)} \frac{3n_0+n_1}{2n_0n_1+4n_0^2}} = 1 + \frac{n_1}{6n_0 + 2n_1} \to \frac{3}{2} \quad \text{as} \quad \frac{n_1}{n_0} \to \infty.$$

For other cases, the theoretical gain can be numerically computed for known n_0 and n_1.

To validate the theoretical derivation, we simulate datasets of 1500 sibling pairs with fixed allele frequency and varying sibling phenotypic correlations and compare the observed relative effective sample size to the theoretical values. From Figure SN1a, we see that the observed values lie almost perfectly on the theoretical trend.

2.1.2 Imputation from parent-offspring pairs

We give the theoretical effective sample size gain for direct effect estimate $\hat{\delta}$ from adding n_1 singletons to a sample of n_0 parent-offspring pairs. Suppose mothers' genotypes are observed fathers' genotypes are imputed using phased data.

Modeling paternal and maternal NTCs First consider Model 2 in the main text with the parameter vector $\begin{bmatrix} \delta & \alpha_p & \alpha_m \end{bmatrix}^\top$. From standard univariate GWAS on the n_1 singletons, we have

$$\hat{\theta}_1 \sim \mathcal{N}\left(\begin{bmatrix} 1 & \frac{1}{2} & \frac{1}{2} \end{bmatrix} \theta, \frac{\sigma_\epsilon^2}{n_1 2f(1-f)}\right). \tag{5}$$

By section 5.3 of the supplementary note in [1], we have

$$\mathrm{Var}(\hat{\theta}_0) = \frac{\sigma_\epsilon^2 + f(1-f)\alpha_p^2}{n_0 f(1-f)} \begin{bmatrix} 2 & -1 & -2 \\ -1 & 1 & 1 \\ -2 & 1 & 3 \end{bmatrix}$$

with

$$\mathrm{Var}(\hat{\delta}_0) = 2\frac{\sigma_\epsilon^2 + f(1-f)\alpha_p^2}{n_0 f(1-f)}.$$

Again by (1),

$$\begin{aligned}\mathrm{Var}(\hat{\theta}) &= \left\{\frac{n_0 f(1-f)}{\sigma_\epsilon^2 + f(1-f)\sigma_p^2} \begin{bmatrix} 2 & -1 & -2 \\ -1 & 1 & 1 \\ -2 & 1 & 3 \end{bmatrix}^{-1} + \frac{2n_1 f(1-f)}{\sigma_\epsilon^2} \begin{bmatrix} 1 & 1 & 1/2 \\ 1/2 & 1/4 & 1/4 \\ 1/2 & 1/4 & 1/4 \end{bmatrix}\right\}^{-1}. \\ &= \frac{\sigma_\epsilon^2 + f(1-f)\alpha_p^2}{2n_0 f(1-f)} \begin{bmatrix} \frac{3\sigma_\epsilon^2 n_1 + 4\sigma_\epsilon^2 n_0 + 3\alpha_p^2 f(1-f) n_1}{\sigma_\epsilon^2 n_1 + \sigma_\epsilon^2 n_0 + \alpha_p^2 f(1-f) n_1} & -2 & -4 \\ -2 & 2 & 2 \\ -4 & 2 & 6 \end{bmatrix}\end{aligned}$$

Figure SN1: Observed and theoretical relative effective sample sizes.

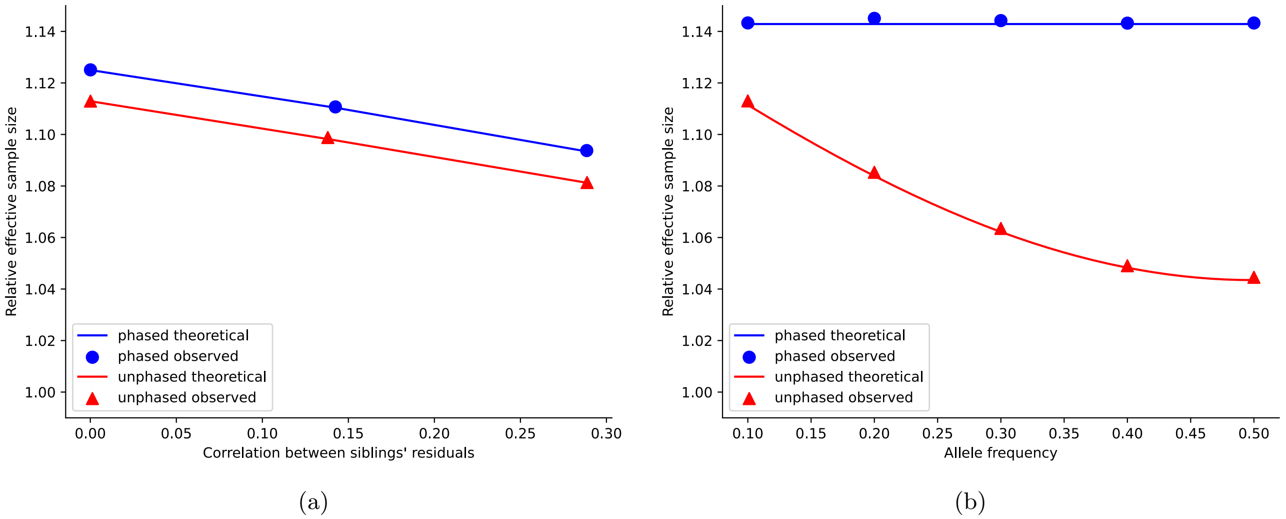

Note. We simulate 3,000 fathers' and 3,000 mothers' genotypes of 1,000 SNPs from independent binomials with 0.5 allele frequency. We then simulate meiosis and produce 3,000 sibling pairs while recording the IBD sharing information (to eliminate the influence of LD structure, we restrict the size of blocks without recombination to 1). Direct SNP effects are simulated from $\mathcal{N}(0, 0.01)$. (a) We randomly remove genotypes of one sibling from 1,500 sibling pairs, resulting in n_0=1,500 genotyped sibling pairs and n_1=1,500 singletons. Parental genotypes of the n_0 sibling pairs are imputed with and without phasing information, and those of the n_1 individuals are linearly imputed. Then we perform two family-based GWAS analyses using n_0 sibling pairs and n_0 sibling pairs plus n_1 individuals respectively, using Model 1 in the main text. Observed relative effective sample sizes (circles and triangles are calculated by taking the ratio of direct effect estimation variance from the n_0 sibling pairs to the estimation variance from the combined sample. The theoretical values are computed using expressions in section 2.1.1. (b) We randomly remove both parents' genotypes of 1,500 offsprings, and fathers' genotypes for the remaining 1,500 offsprings, resulting in n_0=1,500 parent-offspring pairs and n_1=1,500 singletons. Paternal genotypes of the n_0 parent-offspring pairs are imputed with and without phasing information, and both parents' genotypes of the n_1 individuals are linearly imputed. Direct SNP effects are simulated from $\mathcal{N}(0, 0.01)$. Then we perform two family-based GWAS analyses using n_0 sibling pairs and n_0 sibling pairs plus n_1 individuals respectively, modeling direct effect and paternal and maternal NTCs. Observed relative effective sample sizes (circles and triangles are calculated by taking the ratio of direct effect estimation variance from the n_0 parent-offspring pairs to the estimation variance from the combined sample. The theoretical values are computed using expressions in section 2.1.2.

with

$$\mathrm{Var}(\hat{\delta}) = \frac{\sigma_\epsilon^2 + f(1-f)\alpha_p^2}{2n_0 f(1-f)} \frac{3\sigma_\epsilon^2 n_1 + 4\sigma_\epsilon^2 n_0 + 3\alpha_p^2 f(1-f)n_1}{\sigma_\epsilon^2 n_1 + \sigma_\epsilon^2 n_0 + \alpha_p^2 f(1-f)n_1}.$$

Then the relative effective sample size is given by

$$\begin{aligned}\frac{\mathrm{Var}(\hat{\delta}_0)}{\mathrm{Var}(\hat{\delta})} &= \frac{\frac{2\sigma_\epsilon^2 + 2f(1-f)\alpha_p^2}{n_0 f(1-f)}}{\frac{\sigma_\epsilon^2 + f(1-f)\alpha_p^2}{2n_0 f(1-f)} \frac{3\sigma_\epsilon^2 n_1 + 4\sigma_\epsilon^2 n_0 + 3\alpha_p^2 f(1-f)n_1}{\sigma_\epsilon^2 n_1 + \sigma_\epsilon^2 n_0 + \alpha_p^2 f(1-f)n_1}} \\ &= 1 + \frac{\sigma_\epsilon^2 n_1 + \alpha_p^2 f(1-f)n_1}{3\sigma_\epsilon^2 n_1 + 4\sigma_\epsilon^2 n_0 + 3\alpha_p^2 f(1-f)n_1} \\ &= 1 + \frac{n_1 + \frac{\alpha_p^2}{\sigma_\epsilon^2} f(1-f)n_1}{3n_1 + 4n_0 + 3\frac{\alpha_p^2}{\sigma_\epsilon^2} f(1-f)n_1} \to \frac{4}{3} \qquad \text{as} \qquad \frac{n_1}{n_0} \to \infty.\end{aligned}$$

If $\frac{\alpha_p^2}{\sigma_\epsilon^2}$ is close to zero,

$$\frac{\mathrm{Var}(\hat{\delta}_0)}{\mathrm{Var}(\hat{\delta})} \approx 1 + \frac{n_1}{3n_1 + 4n_0}.$$

Modeling average NTCs Now assume $\alpha_p = \alpha_m$ and consider Model 1 in the main text as the true data-generating model (see Appendix 2.2 for a discussion about violation of this assumption). From section 5.3 of the supplementary note in [1], we have

$$\mathrm{Var}(\hat{\theta}_0) = \frac{\sigma_\epsilon^2 + f(1-f)\alpha_p^2}{n_0 f(1-f)} \begin{bmatrix} \frac{3}{2} & -1 \\ -1 & 1 \end{bmatrix}$$

with

$$\mathrm{Var}(\hat{\delta}_0) = \frac{3}{2} \frac{\sigma_\epsilon^2 + f(1-f)\alpha_p^2}{n_0 f(1-f)}.$$

Similarly we have (4) from standard univariate GWAS. Again by (1),

$$\begin{aligned}\text{Var}(\hat{\theta}) &= \left\{ \frac{n_0 f(1-f)}{\sigma_\epsilon^2 + f(1-f)\sigma_p^2} \begin{bmatrix} \frac{3}{2} & -1 \\ -1 & 1 \end{bmatrix}^{-1} + \frac{2n_1 f(1-f)}{\sigma_\epsilon^2} \begin{bmatrix} 1 & 1 \\ 1 & 1 \end{bmatrix} \right\}^{-1}. \\ &= \frac{\sigma_\epsilon^2 + f(1-f)\alpha_p^2}{2n_0 f(1-f)} \begin{bmatrix} \frac{2\sigma_\epsilon^2 n_1 + 3\sigma_\epsilon^2 n_0 + 2\alpha_p^2 f(1-f) n_1}{\sigma_\epsilon^2 n_1 + \sigma_\epsilon^2 n_0 + \alpha_p^2 f(1-f) n_1} & -2 \\ -2 & 2 \end{bmatrix}\end{aligned}$$

with

$$\text{Var}(\hat{\delta}) = \frac{\sigma_\epsilon^2 + f(1-f)\alpha_p^2}{2n_0 f(1-f)} \frac{2\sigma_\epsilon^2 n_1 + 3\sigma_\epsilon^2 n_0 + 2\alpha_p^2 f(1-f) n_1}{\sigma_\epsilon^2 n_1 + \sigma_\epsilon^2 n_0 + \alpha_p^2 f(1-f) n_1}.$$

Then

$$\begin{aligned}\frac{\text{Var}(\hat{\delta}_0)}{\text{Var}(\hat{\delta})} &= \frac{\frac{3\sigma_\epsilon^2 + 3f(1-f)\alpha_p^2}{n_0 f(1-f)}}{\frac{\sigma_\epsilon^2 + f(1-f)\alpha_p^2}{n_0 f(1-f)} \frac{2\sigma_\epsilon^2 n_1 + 3\sigma_\epsilon^2 n_0 + 2\alpha_p^2 f(1-f) n_1}{\sigma_\epsilon^2 n_1 + \sigma_\epsilon^2 n_0 + \alpha_p^2 f(1-f) n_1}} \\ &= 1 + \frac{\sigma_\epsilon^2 n_1 + \alpha_p^2 f(1-f) n_1}{2\sigma_\epsilon^2 n_1 + 3\sigma_\epsilon^2 n_0 + 2\alpha_p^2 f(1-f) n_1} \\ &= 1 + \frac{n_1 + \frac{\alpha_p^2}{\sigma_\epsilon^2} f(1-f) n_1}{2n_1 + 3n_0 + 2\frac{\alpha_p^2}{\sigma_\epsilon^2} f(1-f) n_1} \to \frac{3}{2} \quad \text{as} \quad \frac{n_1}{n_0} \to \infty.\end{aligned}$$

Note that in real-life datasets, α_p^2 is usually trivial relative to σ_ϵ^2. Thus we will have

$$\frac{\text{Var}(\hat{\delta}_0)}{\text{Var}(\hat{\delta})} \approx 1 + \frac{n_1}{2n_1 + 3n_0}.$$

In the case where genotype data is unphased, the effective sample size gain is dependent on the allele frequency and can be computed numerically. We conduct simulations similar to the previous section, and the results are shown in Figure SN1b.

2.2 Modeling average NTC with imputation from parent-offspring pairs in the Young et al. estimator

One somewhat surprising finding is that by assuming $\alpha_p = \alpha_m$ one can get a more precise estimate of δ when analyzing parent-offspring pairs with imputation of the missing parent's genotype. In the case of imputing the missing father's genotype given the mother and offspring's genotype, the portion of variance of the proband's genotype g_{ij} that is uncorrelated with $\hat{g}_{\text{par}(i)} = \hat{g}_{p(i)}$ is larger than the portion uncorrelated with both $\hat{g}_{p(i)}$ and $g_{m(i)}$, and thus we have more information for estimation of δ, at the cost of bias when $\alpha_p \neq \alpha_m$. We establish this result in this section.

Suppose we have n mother-offspring pairs with observed and phased genotypes, g_i and $g_{m(i)}$. Then the father's genotype $g_{p(i)}$ can be imputed the same way as sibling pairs in IBD2:

$$\hat{g}_{p(i)} = \mathbb{E}[\, g_{p(i)} |\, g_i, g_{m(i)}, \text{IBD}_i = 2] = g_i^p + f,$$

where g_i^p is the offspring allele inherited from the father. We model the phenotype using Model 1 in the main text. So we have $\hat{\theta} = \begin{bmatrix} \hat{\delta} & \hat{\alpha} \end{bmatrix}$ and the imputed design matrix $\hat{\mathbf{X}}$ with $[\hat{\mathbf{X}}]_{i\cdot} = \begin{bmatrix} g_i & \hat{g}_{p(i)} + g_{m(i)} \end{bmatrix}$.

If the phenotype is generated by Model 2 in the main text, we have the parameter vector $\theta = \begin{bmatrix} \delta & \alpha_p & \alpha_m \end{bmatrix}$, and the complete design matrix $\mathbf{X}$ with $[\mathbf{X}]_{i\cdot} = \begin{bmatrix} g_i & g_{p(i)} & g_{m(i)} \end{bmatrix}$. Then

$$\text{Cov}([\hat{\mathbf{X}}]_{i:}, [\mathbf{X}]_{i:}) = f(1-f) \begin{bmatrix} 2 & 1 & 1 \\ 2 & 1 & 2 \end{bmatrix}.$$

Therefore,

$$\begin{aligned}
\operatorname*{plim}_{n\to\infty}\hat{\theta} &= \operatorname*{plim}_{n\to\infty}(\hat{\mathbf{X}}^\top\hat{\mathbf{X}})^{-1}\hat{\mathbf{X}}^\top\mathbf{y} \\
&= \operatorname*{plim}_{n\to\infty}(\hat{\mathbf{X}}^\top\hat{\mathbf{X}})^{-1}\hat{\mathbf{X}}^\top\mathbf{X}\theta \\
&= \mathrm{Var}([\hat{\mathbf{X}}]_{i:})^{-1}\mathrm{Cov}([\hat{\mathbf{X}}]_{i:},[\mathbf{X}]_{i:})\theta \\
&= \begin{bmatrix} 1 & \frac{1}{2} & -\frac{1}{2} \\ 0 & 0 & 1 \end{bmatrix}\theta \\
&= \begin{bmatrix} \delta + \frac{1}{2}(\alpha_p - \alpha_m) \\ \alpha_m \end{bmatrix}.
\end{aligned}$$

If $\alpha_p \neq \alpha_m$, the OLS estimate $\hat{\theta}$ is inconsistent and asymptotically biased.

Assume instead $\alpha = \alpha_p = \alpha_m$, then the phenotype generating model is equivalent to Model 1 in the main text. Then $\theta = \begin{bmatrix}\delta & \alpha\end{bmatrix}$ and $[\mathbf{X}]_{i\cdot} = \begin{bmatrix} g_i & g_{p(i)} + g_{m(i)}\end{bmatrix}$. Thus we have

$$\mathrm{Var}([\hat{\mathbf{X}}]_{i:}) = \mathrm{Cov}([\hat{\mathbf{X}}]_{i:},[\mathbf{X}]_{i:}) = f(1-f)\begin{bmatrix} 2 & 2 \\ 2 & 3 \end{bmatrix}.$$

Then,

$$\begin{aligned}
\operatorname*{plim}_{n\to\infty}\hat{\theta} &= \operatorname*{plim}_{n\to\infty}(\hat{\mathbf{X}}^\top\hat{\mathbf{X}})^{-1}\hat{\mathbf{X}}^\top\mathbf{y} \\
&= \operatorname*{plim}_{n\to\infty}(\hat{\mathbf{X}}^\top\hat{\mathbf{X}})^{-1}\hat{\mathbf{X}}^\top\mathbf{X}\theta \\
&= \mathrm{Var}([\hat{\mathbf{X}}]_{i:})\mathrm{Cov}([\hat{\mathbf{X}}]_{i:},[\mathbf{X}]_{i:})\theta \\
&= \theta.
\end{aligned}$$

i.e., the OLS estimate $\hat{\theta}$ is consistent. Also,

$$\begin{aligned}
\mathbb{E}[\hat{\theta}] &= \mathbb{E}[(\hat{\mathbf{X}}^\top\hat{\mathbf{X}})^{-1}\hat{\mathbf{X}}^\top\mathbf{y}] \\
&= \mathbb{E}[(\hat{\mathbf{X}}^\top\hat{\mathbf{X}})^{-1}\hat{\mathbf{X}}^\top\mathbf{X}]\theta \\
&= \mathbb{E}\mathbb{E}[[\,(\hat{\mathbf{X}}^\top\hat{\mathbf{X}})^{-1}\hat{\mathbf{X}}^\top\mathbf{X}|\,g_i, g_{m(i)}, i=1,...,n;\mathrm{IBD}_i=2]]\theta \\
&= \mathbb{E}[(\hat{\mathbf{X}}^\top\hat{\mathbf{X}})^{-1}\hat{\mathbf{X}}^\top\mathbb{E}[\,\mathbf{X}|\,g_i, g_{m(i)}, i=1,...,n;\mathrm{IBD}_i=2]]\theta \\
&= \mathbb{E}[(\hat{\mathbf{X}}^\top\hat{\mathbf{X}})^{-1}\hat{\mathbf{X}}^\top\hat{\mathbf{X}}]\theta \\
&= \theta,
\end{aligned}$$

i.e., $\hat{\theta}$ is unbiased. By the supplementary note in [1], we have

$$\mathrm{Var}(\hat{\theta}) = \frac{\sigma_\epsilon^2 + f(1-f)\alpha_p^2}{nf(1-f)}\begin{bmatrix} \frac{3}{2} & -1 \\ -1 & 1 \end{bmatrix},$$

with

$$\mathrm{Var}(\hat{\delta}) = \frac{3}{2}\frac{\sigma_\epsilon^2 + f(1-f)\alpha_p^2}{nf(1-f)}.$$

For $\theta_c = \begin{bmatrix}\delta & \alpha_p & \alpha_m\end{bmatrix}$, we have

$$\mathrm{Var}(\hat{\delta}_c) = 2\frac{\sigma_\epsilon^2 + f(1-f)\alpha_p^2}{nf(1-f)},$$

and $\mathrm{Var}(\hat{\delta}) < \mathrm{Var}(\hat{\delta}_c)$.

2.3 The non-transmitted estimator

2.3.1 Effect of population structure

In the main text we claimed that the non-transmitted estimator is immune to population structure. Before giving a derivation, we first examine the effect of population structure on imputation and different family-based and standard GWAS estimators. As in [1], we consider an island model of population structure: the population is divided into K subpopulations, with random-mating within each subpopulation and no migration between them. For one locus, we denote the kth subpopulation allele

frequency as f_k for $k = 1, 2, \ldots, K$, and the overall population allele frequency as $f = \mathbb{E}_k[f_k]$, with the expectation taken over the K subpopulations. Parental genotype imputation that we have been considering so far relies on the overall population allele frequency. In the presence of population structure, imputation based on the overall population allele frequency f will be biased if f_k's are different from f. As a result, bias might also be introduced into the direct effect estimate. For instance, for imputation from a sibling pair with phased data, the imputed sum of parental genotypes for family i in subpopulation k is given by

$$\hat{g}_{k\text{par}(i)} = \begin{cases} g_{k\text{par}(i)} & \text{IBD}_{ki} = 0; \\ g_{ki1} + g'_{ki2} + f & \text{IBD}_{ki} = 1; \\ g_{ki1} + 2f & \text{IBD}_{ki} = 2, \end{cases}$$

where g'_{ki2} is the allele of sibling 2 that is not shared IBD with alleles inherited by sibling 1, and IBD_{ki} is the IBD state of the ith sibling pair in the kth subpopulation. Taking the expectation, we have $\mathbb{E}[\hat{g}_{k\text{par}(i)}] = 3f_k + f \neq 4f_k$. Note that we also have $\mathbb{E}[\,\hat{g}_{k\text{par}(i)}\,|\,\text{IBD}_{ki} = 0] = 4f_k$, $\mathbb{E}[\,\hat{g}_{k\text{par}(i)}\,|\,\text{IBD}_{ki} = 1] = 3f_k + f$, and $\mathbb{E}[\,\hat{g}_{k\text{par}(i)}\,|\,\text{IBD}_{ki} = 2] = 2f_k + 2f$. The variation in the expectation of the imputed parental genotypes across IBD states is due to the difference in the number of observed parental alleles.

Consider performing family-based GWAS using siblings with parental genotypes imputed as above and with the following phenotype model:

$$Y_{kij} = \delta g_{kij} + \alpha g_{k\text{par}(i)} + \epsilon_{kij}, \tag{6}$$

where Y_{kij} is the phenotype of sibling j in family i from subpopulation k, and ϵ_{kij} is uncorrelated with both proband and parental genotype. Young et al. [1] showed that performing the regression with $g_{k\text{par}(i)}$ replaced with $\hat{g}_{k\text{par}(i)}$ (as defined above) gives, in limit, $\text{plim}_{n\to\infty}\,\hat{\delta} \to \delta + c\alpha$, where c is a function of Wright's F_{st}, defined as $F_{st} = \text{Var}_k(f_k)/[f(1-f)]$ in this model. When F_{st} is small, $c \approx F_{st}/2$, implying the bias will be negligible for European genetic ancestry samples, where F_{st} has been estimated to be on the order of 10^{-3}[3]. In contrast, under this model of population structure, the population effect is $\beta = \delta + \frac{1+3F_{st}}{1+F_{st}}\alpha$.

2.3.2 Properties of the non-transmitted estimator

We now provide a derivation of the consistency and asymptotic unbiasedness of the non-transmitted estimator in the aforementioned island model of population structure. We proceed by first proving the claim for each group in Table 1 in the main text. For 'paternal NT', 'maternal NT', and 'both NT', we consider a single proband from each family, while for 'one NT', we consider sibling pairs. n_{pat}, n_{mat}, n_{both} and n_{one} are the numbers of families in the four groups, and families are assumed to be independent. The fully observed and imputed design matrices are given by $\mathbf{X}$ and $\hat{\mathbf{X}}$ with the corresponding subscripts. We denote the parameter vectors for the 'paternal NT' and 'maternal NT' groups are $\theta = \begin{bmatrix}\delta & \alpha_p & \alpha_m\end{bmatrix}$, and the parameter vectors for the 'both NT' and 'one NT' groups are $\theta = \begin{bmatrix}\delta & \alpha\end{bmatrix}$. In table SN2, we list the limiting statistics related to estimations in the four groups, which are used to prove the claimed properties; and we give the sampling variances of the resulting estimators in each group.

We start by considering 'both NT', that is, the situation where both non-transmitted parental alleles are known. As all four parental alleles have been observed, the sum and imputed sum of the parental genotypes are the same: $\hat{g}_{\text{par}(i)} = g_{\text{par}(i)}$, and the true and imputed design matrices coincide: $\hat{\mathbf{X}}_{\text{both}} = \mathbf{X}_{\text{both}}$, where $[\mathbf{X}_{\text{both}}]_{i:} = \begin{bmatrix}g_i & g_{\text{par}(i)}\end{bmatrix}$. Denote the effect estimate by $\hat{\theta}_{\text{both}}$. Then

$$\begin{aligned}
\underset{n\to\infty}{\text{plim}}\,\hat{\theta}_{\text{both}} &= \underset{n\to\infty}{\text{plim}}\,(\hat{\mathbf{X}}_{\text{both}}^\top \hat{\mathbf{X}}_{\text{both}})^{-1}\hat{\mathbf{X}}_{\text{both}}^\top \mathbf{y} \\
&= \underset{n\to\infty}{\text{plim}}\,(\hat{\mathbf{X}}_{\text{both}}^\top \hat{\mathbf{X}}_{\text{both}})^{-1}\hat{\mathbf{X}}_{\text{both}}^\top \mathbf{X}_{\text{both}}\theta \\
&= \underset{n\to\infty}{\text{plim}}\,(\mathbf{X}_{\text{both}}^\top \mathbf{X}_{\text{both}})^{-1}\mathbf{X}_{\text{both}}^\top \mathbf{X}_{\text{both}}\theta \\
&= \theta = \begin{bmatrix}\delta \\ \alpha\end{bmatrix},
\end{aligned}$$

and specifically, the direct effect estimate $\hat{\delta}_{\text{both}}$ is consistent and asymptotically unbiased.

Next we look at 'maternal NT', the scenario where the non-transmitted maternal allele is observed and but the paternal one is not ('Maternal NT' in Table 1 in the main text. In the opposite situation 'paternal NT', the claim will follow by symmetry. In this case, the mother's genotypes are completely determined: $\hat{g}_{m(i)} = g_{m(i)}$. Then the imputed and complete design matrices are $\hat{\mathbf{X}}_{\text{mat}}$ and $\mathbf{X}_{\text{mat}}$ respectively, with $[\hat{\mathbf{X}}_{\text{mat}}]_{i:} = \begin{bmatrix}g_i & \hat{g}_{p(i)} & g_{m(i)}\end{bmatrix}$ and $[\mathbf{X}_{\text{mat}}]_{i:} = \begin{bmatrix}g_i & g_{p(i)} & g_{m(i)}\end{bmatrix}$. For the corresponding effect estimate $\hat{\theta}_{\text{mat}}$ with paternal non-transmitted alleles imputed, we follow the supplementary note in Young et al. [1] and obtain (with a minor correction)

$$
\begin{aligned}
\operatorname*{plim}_{n_{\text{mat}}\to\infty} \hat{\theta}_{\text{mat}} &= \operatorname*{plim}_{n_{\text{mat}}\to\infty} (\hat{\mathbf{X}}_{\text{mat}}^\top \hat{\mathbf{X}}_{\text{mat}})^{-1} \mathbf{X}_{\text{mat}}^\top \mathbf{y} \\
&= \operatorname*{plim}_{n_{\text{mat}}\to\infty} (\hat{\mathbf{X}}_{\text{mat}}^\top \hat{\mathbf{X}}_{\text{mat}})^{-1} \mathbf{X}_{\text{mat}}^\top [\mathbf{X}_{\text{mat}}]_{i:}\theta \\
&= \text{Var}([\hat{\mathbf{X}}_{\text{mat}}]_{i:}) \text{Cov}([\hat{\mathbf{X}}_{\text{mat}}]_{i:}, [\mathbf{X}_{\text{mat}}]_{i:})\theta \\
&= \begin{bmatrix} 2(1+F_{\text{st}}) & 1+F_{\text{st}} & 1+3F_{\text{st}} \\ 1+F_{\text{st}} & 1 & 2F_{\text{st}} \\ 1+3F_{\text{st}} & 2F_{\text{st}} & 2(1+F_{\text{st}}) \end{bmatrix}^\top \\
&\qquad \cdot \begin{bmatrix} 2(1+F_{\text{st}}) & 1+3F_{\text{st}} & 1+3F_{\text{st}} \\ 1+F_{\text{st}} & 1+F_{\text{st}} & 2F_{\text{st}} \\ 1+3F_{\text{st}} & 4F_{\text{st}} & 2(1+F_{\text{st}}) \end{bmatrix} \theta \\
&= \begin{bmatrix} \delta \\ (1+c)\alpha_p \\ \alpha_m + c\alpha_p \end{bmatrix}, \text{ where } c = \frac{F_{\text{st}}}{1+2F_{\text{st}}}.
\end{aligned}
$$

Therefore, the direct effect estimate $\hat{\delta}_{\text{mat}}$ is consistent and asymptotically unbiased, although the non-transmitted coefficient estimates are biased. Similarly for 'paternal NT', we have

$$
\operatorname*{plim}_{n_{\text{pat}}\to\infty} \hat{\theta}_{\text{pat}} = \begin{bmatrix} \delta \\ c\alpha_m + \alpha_p \\ (1+c)\alpha_m \end{bmatrix},
$$

establishing consistency and asymptotic unbiasedness of $\hat{\delta}_{\text{pat}}$.

Young et al. [1] show that the direct effect estimate $\hat{\delta}_{\text{one}}$ from sibling pairs in IBD1 (in fact, all possible cases in group 'one NT' in Table 1 in the main text collapse to a sibling pair in IBD1) is also consistent and asymptotically unbiased:

$$
\operatorname*{plim}_{n_{\text{one}}\to\infty} \hat{\theta}_{\text{one}} = \begin{bmatrix} \delta \\ \frac{1+3F_{\text{st}}}{1+2F_{\text{st}}}\alpha \end{bmatrix}.
$$

As is shown in (2.1), the non-transmitted estimator, which analyze the combined sample of the four groups in Table 1 in the main text, is equivalent to meta-analyzing $\hat{\delta}_{\text{both}}$, $\hat{\delta}_{\text{mat}}$, $\hat{\delta}_{\text{pat}}$ and $\hat{\delta}_{\text{one}}$. As these four estimators are consistent and asymptotically unbiased, so is the non-transmitted estimator.

Table SN2: Limiting statistics for the four groups in Table 1 in the main text.

Group	$\text{Var}([\hat{\mathbf{X}}]_{i:})$	$\text{Cov}([\hat{\mathbf{X}}]_{i:}, [\mathbf{X}]_{i:})$
Maternal NT	$f(1-f)\begin{bmatrix} 2(1+F_{\text{st}}) & 1+F_{\text{st}} & 1+3F_{\text{st}} \\ 1+F_{\text{st}} & 1 & 2F_{\text{st}} \\ 1+3F_{\text{st}} & 2F_{\text{st}} & 2(1+F_{\text{st}}) \end{bmatrix}$	$f(1-f)\begin{bmatrix} 2(1+F_{\text{st}}) & 1+3F_{\text{st}} & 1+3F_{\text{st}} \\ 1+F_{\text{st}} & 1+F_{\text{st}} & 2F_{\text{st}} \\ 1+3F_{\text{st}} & 4F_{\text{st}} & 2(1+F_{\text{st}}) \end{bmatrix}$
Paternal NT	$f(1-f)\begin{bmatrix} 2(1+F_{\text{st}}) & 1+3F_{\text{st}} & 1+F_{\text{st}} \\ 1+3F_{\text{st}} & 2(1+F_{\text{st}}) & 2F_{\text{st}} \\ 1+F_{\text{st}} & 2F_{\text{st}} & 1 \end{bmatrix}$	$f(1-f)\begin{bmatrix} 2(1+F_{\text{st}}) & 1+3F_{\text{st}} & 1+3F_{\text{st}} \\ 1+3F_{\text{st}} & 2(1+F_{\text{st}}) & 4F_{\text{st}} \\ 1+F_{\text{st}} & 2F_{\text{st}} & 1+F_{\text{st}} \end{bmatrix}$
Both NT	$f(1-f)\begin{bmatrix} 2(1+F_{\text{st}}) & 2(1+3F_{\text{st}}) \\ 2(1+3F_{\text{st}}) & 4F_{\text{st}} \end{bmatrix}$	$f(1-f)\begin{bmatrix} 2(1+F_{\text{st}}) & 2(1+3F_{\text{st}}) \\ 2(1+3F_{\text{st}}) & 4F_{\text{st}} \end{bmatrix}$
One NT	$\begin{bmatrix} (2-r)+(2-3r)F_{\text{st}} & 2(1-r)(1+2F_{\text{st}}) \\ 2(1-r)(1+2F_{\text{st}}) & 3(1-r)(1+2F_{\text{st}}) \end{bmatrix}$	$f(1-f)\begin{bmatrix} (2-r)+(2-3r)F_{\text{st}} & 2(1-r)(1+3F_{\text{st}}) \\ 2(1-r)(1+2F_{\text{st}}) & 3(1-r)(1+3F_{\text{st}}) \end{bmatrix}$

Group	$\text{Var}(\hat{\theta})$	$\text{Var}(\hat{\delta})$
Maternal NT	$\frac{\sigma_\epsilon^2}{2n_{\text{pat}}f(1-f)(1-F_{\text{st}})}\begin{bmatrix} 2 & -2 & -1 \\ -2 & \frac{5F_{\text{st}}+3}{2F_{\text{st}}+1} & \frac{F_{\text{st}}+1}{2F_{\text{st}}+1} \\ -1 & \frac{F_{\text{st}}+1}{2F_{\text{st}}+1} & \frac{F_{\text{st}}+1}{2F_{\text{st}}+1} \end{bmatrix}$	$\frac{\sigma_\epsilon^2}{n_{\text{pat}}f(1-f)(1-F_{\text{st}})}$
Paternal NT	$\frac{\sigma_\epsilon^2}{2n_{\text{mat}}f(1-f)(1-F_{\text{st}})}\begin{bmatrix} 2 & -1 & -2 \\ -1 & \frac{F_{\text{st}}+1}{2F_{\text{st}}+1} & \frac{F_{\text{st}}+1}{2F_{\text{st}}+1} \\ -2 & \frac{F_{\text{st}}+1}{2F_{\text{st}}+1} & \frac{5F_{\text{st}}+3}{2F_{\text{st}}+1} \end{bmatrix}$	$\frac{\sigma_\epsilon^2}{n_{\text{mat}}f(1-f)(1-F_{\text{st}})}$
Both NT	$\frac{\sigma_\epsilon^2}{n_{\text{both}}f(1-f)(1-F_{\text{st}})}\begin{bmatrix} 2 & -1 \\ -1 & \frac{F_{\text{st}}-1}{3F_{\text{st}}+1} \end{bmatrix}$	$\frac{\sigma_\epsilon^2}{2n_{\text{both}}f(1-f)(1-F_{\text{st}})}$
One NT	$\frac{(1+r)\sigma_\epsilon^2}{2n_{\text{one}}(2+r)(1-F_{\text{st}})(1+2F_{\text{st}})f(1-f)}\begin{bmatrix} 3(1-r)(1+2F_{\text{st}}) & -2(1-r)(1+2F_{\text{st}}) \\ -2(1-r)(1+2F_{\text{st}}) & (2-r)+(2-3r)F_{\text{st}} \end{bmatrix}$	$\frac{3(1-r^2)\sigma_\epsilon^2}{2n_{\text{one}}(2+r)f(1-f)(1-F_{\text{st}})}$

2.3.3 Effect of admixture

Although the non-transmitted estimator is robust to population structure, such robustness does not hold when the method is applied to admixed samples. We analyze the effect of sample admixture in this section. We first assume the Pritchard, Stephens, and Donnelly's admixture model [4]: for each SNP, we posit that for each individual, each of their 4 parental alleles on a locus is independently sampled from a mixture of K allele frequencies denoted by $f_1, f_2, \dots, f_K$, with mean $\mathbb{E}[f_k] = f$ and Wright's $F_{\text{st}} = \frac{\text{Var}(f_k)}{f(1-f)}$ for $k = 1, \dots, K$. That is, for each individual's paternally and maternally inherited alleles g^f and g^m, we have

$$g^f \overset{d}{=} g^m \sim \sum_{k=1}^{K} \pi_i \text{Bern}(f_k), \qquad \sum_{k=1}^{K} \pi_k = 1.$$

Note that

$$\mathbb{E}[g^f] = \mathbb{E}[g^m] = f$$

and

$$\text{Var}(g^f) = \text{Var}(g^m) = \mathbb{E}[\text{Var}(g^f)|k] + \text{Var}(f_k) = f(1-f),$$

where f is the population allele frequency. To show if the non-transmitted estimator is consistent or not, it suffices to consider its consistency in each of the four groups. In this section, we look at the 'one NT' case. Now observe that for a pair of siblings sharing one allele IBD in the ith family, we have

$$\text{Var}(g_{i1}) = \text{Var}(g_{i2}) = 2f(1-f)$$

and

$$\begin{aligned}
\text{Cov}(g_{i1}, g_{i2} \mid \text{IBD} = 1) &= \text{Cov}(g_{i1}^p + g^q, g_{i2}^p + g^q) \\
&= \mathbb{E}_{p \neq q \in \{f,m\}}[\text{Var}(g^q)] + \text{Var}_{p \neq q \in \{f,m\}}(2f) \\
&= f(1-f).
\end{aligned}$$

Furthermore,

$$\begin{aligned}
\text{Cov}(\hat{g}_{\text{par}(i)}, g_{i1} + g_{i2} \mid \text{IBD} = 1) &= \text{Cov}(g_{i1}^p + g_{i2}^p + g^q + f, g_{i1}^p + g_{i2}^p + 2g^q) \\
&= \mathbb{E}_{p \neq q \in \{f,m\}}[\text{Var}(g_{i1}^p) + \text{Var}(g_{i2}^p) + 2\text{Var}(g^q)] + \text{Cov}_{p \neq q \in \{f,m\}}(4f, 4f) \\
&= 4f(1-f),
\end{aligned}$$

$$\begin{aligned}
\text{Var}(\hat{g}_{\text{par}(i)} \mid \text{IBD} = 1) &= \text{Var}(g_{i1}^p + g_{i2}^p + g^q + f) \\
&= \mathbb{E}_{p \neq q \in \{f,m\}}[\text{Var}(g_{i1}^p) + \text{Var}(g_{i2}^p) + \text{Var}(g^q)] + \text{Var}_{p \neq q \in \{f,m\}}(4f) \\
&= 3f(1-f),
\end{aligned}$$

$$\begin{aligned}
\text{Cov}(g_{i1}, g_{\text{par}(i)} \mid \text{IBD} = 1) &= \text{Cov}(g_{i1}^p + g^q, g_{i1}^p + g_{i2}^p + g^q + g_0^q) \\
&= \mathbb{E}_{p \neq q \in \{f,m\}}[\text{Var}(g_{i1}^p) + \text{Var}(g^q)] + \text{Cov}_{p \neq q \in \{f,m\}}(2f, 4f) \\
&= 2f(1-f),
\end{aligned}$$

and

$$\begin{aligned}
\text{Cov}(\hat{g}_{\text{par}(i)}, g_{\text{par}(i)} \mid \text{IBD} = 1) &= \text{Cov}(g_{i1}^p + g_{i2}^p + g^q + f, g_{i1}^p + g_{i2}^p + g^q + g_0^q) \\
&= \mathbb{E}_{p \neq q \in \{f,m\}}[\text{Var}(g_{i1}^p) + \text{Var}(g_{i2}^p) + \text{Var}(g^q)] + \text{Cov}_{p \neq q \in \{f,m\}}(4f, 4f) \\
&= 3f(1-f),
\end{aligned}$$

where g_0^q is the non-transmitted parental allele. Then

$$\sum_i \mathbf{X}_i^\top \mathbf{\Sigma}_i^\top \mathbf{X}_i \to \frac{2n_1 f(1-f)}{\sigma_\epsilon^2(1-r^2)} \begin{bmatrix} 1-r & 2(1-r) \\ 2(1-r) & 3(1-r) \end{bmatrix}$$

and

$$\sum_i \mathbf{X}_i^\top \mathbf{\Sigma}_i^\top \mathbf{y}_i \to \frac{2n_1 f(1-f)}{\sigma_\epsilon^2(1-r^2)} \begin{bmatrix} 1-r & 2(1-r) \\ 2(1-r) & 3(1-r) \end{bmatrix},$$

where r is the correlation of sibling residuals. If the true phenotype-generating model is

$$y_{ij} = \delta g_i + \alpha g_{\text{par}(i)} + \epsilon_{ij}$$

for the jth sibling in the ith family, we then have

$$\hat{\theta} \to \begin{bmatrix} \delta \\ \alpha \end{bmatrix},$$

which means $\hat{\delta}$ is consistent.

A more general and perhaps more realistic case is where the father's alleles are drawn from a mixture of K_1 allele frequencies $f_1^f, \ldots, f_{K_1}^f$ with F_{st}^f with $\mathbb{E}[f_k^f] = f^f$, and the mother's alleles are independently drawn from a different mixture of K_2 allele frequencies $f_1^m, \ldots, f_{K_2}^m$ with F_{st}^m and $\mathbb{E}[f_k^m] = f^m$. That is, we have

$$g^f \sim \sum_{k=1}^{K_1} \pi_i^f \text{Bern}(f_k^f), \qquad \sum_{k=1}^{K_1} \pi_k^f = 1,$$

and

$$g^m \sim \sum_{k=1}^{K_2} \pi_i^m \text{Bern}(f_k^m), \qquad \sum_{k=1}^{K_2} \pi_k^m = 1,$$

and g^f and g^m are independent. Denote the variance of the mean allele frequency by

$$z \triangleq \text{Var}_{p\in\{f,m\}}(f^p) = \frac{1}{2}[(f^f)^2 + (f^m)^2] - f^2, \qquad \text{where} \quad f = \frac{1}{2}(f^f + f^m).$$

We follow this setting and obtain

$$\text{Var}(g^f) = f^f(1 - f^f)$$

and

$$\text{Var}(g^m) = f^m(1 - f^m).$$

We define

$$u \triangleq \text{Var}(g) = \text{Var}(g^f) + \text{Var}(g^m).$$

Then

$$\begin{aligned}
\text{Cov}(g_{i1}, g_{i2} \mid \text{IBD} = 1) &= \mathbb{E}_{p\neq q}[\text{Cov}(g_{i1}^q + g^p, g_{i2}^q + g^p)] + \text{Cov}_{p\neq q}(f^p + f^q, f^p + f^q) \\
&= \frac{1}{2}(\text{Var}(g^f) + \text{Var}(g^m)] + \text{Cov}_{p\neq q}(2f, 2f) \\
&= \frac{1}{2}u,
\end{aligned}$$

$$\begin{aligned}
\text{Cov}(\hat{g}_{\text{par}(i)}, g_{i1} + g_{i2} \mid \text{IBD} = 1) &= \mathbb{E}_{p\neq q}[\text{Cov}(g_{i1}^q + g_{i2}^q + g^p + f, g_{i1}^q + g_{i2}^q + 2g^p)] \\
&\quad + \text{Cov}_{p\neq q}(2f^q + f^p, 2f^p + 2f^q) \\
&= 2[\text{Var}(g^f) + \text{Var}(g^m)] + \text{Cov}_{p\neq q}(2f^p + f^q, 4f) \\
&= 2u,
\end{aligned}$$

$$\begin{aligned}
\text{Var}(\hat{g}_{\text{par}(i)} \mid \text{IBD} = 1) &= \mathbb{E}_{p\neq q}[\text{Var}(g_{i1}^q + g_{i2}^q + g^p + f)] \\
&\quad + \text{Var}_{p\neq q}(2f^q + f^p) \\
&= \frac{3}{2}[\text{Var}(g^f) + \text{Var}(g^m)] + \text{Var}_{p\neq q}(f^p + 2f) \\
&= \frac{3}{2}u + \text{Var}(f^p) \\
&= \frac{3}{2}u + \frac{1}{2}((f^f)^2 + (f^m)^2) - f^2 \\
&= \frac{3}{2}u + z,
\end{aligned}$$

$$\begin{aligned}
\text{Cov}(\hat{g}_{\text{par}(i)}, g_{\text{par}(i)} \mid \text{IBD} = 1) &= \mathbb{E}_{p\neq q}[\text{Cov}(g_{i1}^q + g_{i2}^q + g^p + f, g_{i1}^q + g_{i2}^q + g^p + g_0^p)] \\
&\quad + \text{Cov}_{p\neq q}(2f^p + f^q, 2f^p + 2f^q)) \\
&= \frac{3}{2}[\text{Var}(g^f) + \text{Var}(g^m)] + \text{Cov}_{p\neq q}(2f^p + f^q, 4f) \\
&= \frac{3}{2}u,
\end{aligned}$$

and

$$\begin{aligned}\mathrm{Cov}(g_{\mathrm{par}(i)}, g_{i1} \mid \mathrm{IBD}=1) &= \mathbb{E}_{p\neq q}[\mathrm{Cov}(g_{i1}^q + g_{i2}^q + g^p + g_0^p, g_{i1}^q + g^p)] \\ &\quad + \mathrm{Cov}_{p\neq q}(2f^p + 2f^q, f^p + f^q)) \\ &= \mathrm{Var}(g^f) + \mathrm{Var}(g^m) + \mathrm{Cov}_{p\neq q}(4f, 2f) \\ &= u.\end{aligned}$$

Then

$$\sum_i \mathbf{X}_i^\top \boldsymbol{\Sigma}_i^\top \mathbf{X}_i \to \frac{2n_1}{\sigma_\epsilon^2(1-r^2)} \begin{bmatrix} u(1-\frac{1}{2}r) & u(1-r) \\ u(1-r) & (\frac{3}{2}u + z)(1-r) \end{bmatrix}$$

and

$$\sum_i \mathbf{X}_i^\top \boldsymbol{\Sigma}_i^\top \mathbf{y}_i \to \frac{2n_1}{\sigma_\epsilon^2(1-r^2)} \begin{bmatrix} u(1-\frac{1}{2}r) & u(1-r) \\ u(1-r) & \frac{3}{2}u(1-r) \end{bmatrix}.$$

It follows that

$$\hat{\theta} \to \begin{bmatrix} 1 & \frac{4z(1-r)}{u(2+r)+2z(2-r)} \\ 0 & \frac{u(r+2)}{u(2+r)+2z(2-r)} \end{bmatrix} \begin{bmatrix} \delta \\ \alpha \end{bmatrix},$$

i.e., $\hat{\theta}$ is inconsistent in general.

We formulate the bias factor as a function of the squared distance between the paternal and maternal mean allele frequencies $d = |f^f - f^g|$. Then

$$z = \frac{1}{2}[(f-d)^2 + (f+d)^2] - f^2 = d^2,$$

and

$$u = (f-d)(1-(f-d)) + (f+d)(1-(f+d)) = 2[f(1-f) - d^2].$$

So the bias factor is

$$\frac{4z(1-r)}{u(2+r)+2z(2-r)} = \frac{4d^2(1-r)}{2[f(1-f)-d^2](2+r)+2d^2(2-r)}.$$

We conducted a simulation study to evaluate the bias in the more general case. We simulated 20,000 families (2,000 sibling pairs and 18,000 singletons) and 20,000 SNPs, where the paternal and maternal genotypes are sampled independently. For the paternal side, we first simulated a 20,000-by-2 matrix of allele frequencies: $[\mathbf{f}_A^f \quad \mathbf{f}_B^f]$, in the sth row of which the two allele frequencies $f_{A;s}^f$ and $f_{B;s}^f$ were drawn from a Balding-Nichols model with mean allele frequency $f^f = 0.7$. In each family, we randomly assigned the father to either column A or B, and his genotypes were then sampled according to $\mathbf{f}_A^f$ or $\mathbf{f}_B^f$. Maternal genotypes were simulated in a similar fashion with respect to the matrix $[\mathbf{f}_C^m \quad \mathbf{f}_D^m]$ but with mean allele frequency $f^m = 0.3$. For this simulation study, we assume $F_{\mathrm{st}}^f = F_{\mathrm{st}}^m$. With the above setting, the fathers and mothers were assigned to the groups A or B, and C or D. We considered the group means $y_A = -1$, $y_B = 1$, $y_C = -2$ and $y_D = 2$. The phenotype of the jth sibling in the ith family is defined as

$$y_{ij} = \frac{1}{2}[(\mathbb{1}_{\{\text{father is in } A\}} y_A + \mathbb{1}_{\{\text{father is in } B\}} y_B) + (\mathbb{1}_{\{\text{mother is in } C\}} y_C + \mathbb{1}_{\{\text{mother is in } D\}} y_D)] + \epsilon_{ij},$$

where ϵ_{ij} are scaled to take up 50% of the variance of y_{ij}. We applied the non-transmitted estimator, the sib-difference method, the Young et al. estimator, and the unified estimator to the simulated datasets with varying degrees of F_{st} and obtained direct effect estimates. As in the main text, we examine the non-sampling variances relative to that from the standard GWAS with $F_{\mathrm{st}} = 0.001$, as well as the variance of Z^2. From Figure SN2, we see that when $F_{\mathrm{st}} = 0.001$, none of the four methods show detectable bias. As F_{st} increases, the sib-difference method remains free of bias; however, although not as strong as that of the Young et al. estimator, the non-transmitted estimator indeed shows increasing bias as F_{st} increases.

2.4 The robust estimator

As for the non-transmitted estimator, the sample is also partitioned into four groups based on which non-transmitted alleles are observed for the robust estimator. For the 'both NT' group, we perform family-based regressions as before. for the 'maternal NT' and 'paternal NT' groups, we perform uniparental regressions: that is, we fit

$$y_{ij} \sim g_{ij}^m + g_{m(i)}$$

for 'maternal NT' and

$$y_{ij} \sim g_{ij}^p + g_{p(i)}$$

Figure SN2: Bias in direct effect estimates obtained from admixed samples.

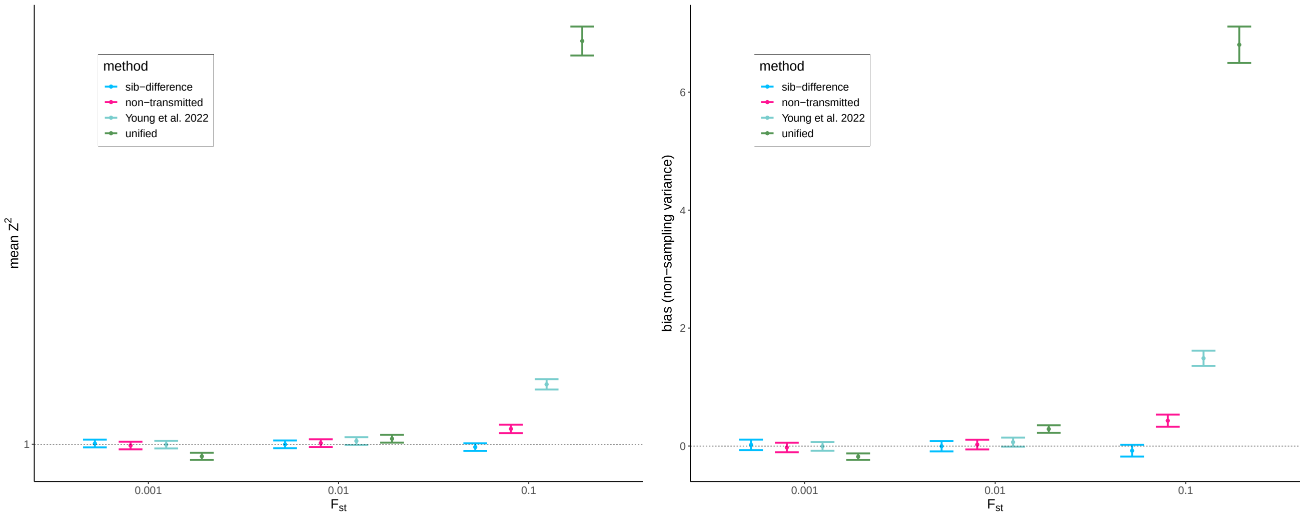

(a) Mean Z^2.

(b) Non-sampling variance.

Note. We simulated 20,000 families (2,000 sibling pairs and 18,000 singletons) and 20,000 SNPs. (a) Mean of Z^2 statistics across 20,000 SNPs for the four estimators, which are expected to be above 1 (dashed-line) when there is unaccounted-for confounding; (b) mean of non-sampling variance (see Equation 2 in the Methods section in the main text) of the estimators relative to the that observed for standard GWAS with $F_{\mathrm{st}} = 0.001$, which gives a measure of the magnitude of bias due to population stratification, with values above 0 indicating bias. Error bars display 95% jackknife confidence intervals over 20,000 SNPs.

for 'paternal NT', where g_{ij}^m and g_{ij}^p are the paternal and maternal transmitted alleles, respectively. Given the genotypes of the observed parent, the transmitted alleles from that parent are due to random segregation during meioses, so the direct effect estimate is unbiased even in the presence of population stratification or sample admixture. For the 'one NT' group, we perform sib-difference regressions as in Section 2.5, which is also robust to population structure and sample admixture. Thus, the overall robustness of the robust estimator follows from the argument through meta-analysis.

2.4.1 Implementation of the robust estimator

In the implementation of the robust estimator in *snipar*, a generalized least square (GLS) is used for each group, where the precision matrix is the inverse of the corresponding block of the $\mathbf{V}$ matrix from the LMM. For example, for the 'both NT' group, the effect estimate is given by

$$\hat{\theta}_{\mathrm{both}} = (\hat{\mathbf{X}}_{\mathrm{both}}^{\top}\mathbf{V}_{\mathrm{both}}^{-1}\hat{\mathbf{X}}_{\mathrm{both}})^{-1}\hat{\mathbf{X}}_{\mathrm{both}}^{\top}\mathbf{V}_{\mathrm{both}}^{-1}\mathbf{y}_{\mathrm{both}},$$

and the direct effect estimate $\hat{\delta}_{\mathrm{both}}$ is simply the first entry of this vector. The estimates from the other groups: $\hat{\delta}_{\mathrm{mat}}$, $\hat{\delta}_{\mathrm{pat}}$ and $\hat{\delta}_{\mathrm{one}}$ are similarly obtained, except that we have $[\hat{\mathbf{X}}_{\mathrm{mat}}]_{i:} = \begin{bmatrix} g_i^m & g_{m(i)} \end{bmatrix}$ for 'maternal NT', $[\hat{\mathbf{X}}_{\mathrm{pat}}]_{i:} = \begin{bmatrix} g_i^p & g_{p(i)} \end{bmatrix}$ for 'paternal NT' and $[\hat{\mathbf{X}}_{\mathrm{one}}]_{i:} = \begin{bmatrix} g_i & g_{s(i)} \end{bmatrix}$ for 'one NT'. Following [5], the robust estimator for the direct effect is given by

$$\hat{\delta}_{\mathrm{robust}} = (\mathbf{A}^{\top}\mathbf{S}^{-1}\mathbf{A})^{\top}\mathbf{A}^{\top}\mathbf{S}^{-1}\begin{bmatrix}\hat{\delta}_{\mathrm{both}} & \hat{\delta}_{\mathrm{one}} & \hat{\delta}_{\mathrm{pat}} & \hat{\delta}_{\mathrm{mat}}\end{bmatrix}^{\top},$$

where

$$\mathbf{A} = \begin{bmatrix}1\\1\\1\\1\end{bmatrix} \quad \text{and} \quad \mathbf{S} = \begin{bmatrix} \mathrm{Var}(\hat{\delta}_{\mathrm{both}}) & \mathrm{Cov}(\hat{\delta}_{\mathrm{both}}, \hat{\delta}_{\mathrm{one}}) & \mathrm{Cov}(\hat{\delta}_{\mathrm{both}}, \hat{\delta}_{\mathrm{pat}}) & \mathrm{Cov}(\hat{\delta}_{\mathrm{both}}, \hat{\delta}_{\mathrm{mat}}) \\ \mathrm{Cov}(\hat{\delta}_{\mathrm{both}}, \hat{\delta}_{\mathrm{one}}) & \mathrm{Var}(\hat{\delta}_{\mathrm{one}}) & \mathrm{Cov}(\hat{\delta}_{\mathrm{one}}, \hat{\delta}_{\mathrm{pat}}) & \mathrm{Cov}(\hat{\delta}_{\mathrm{one}}, \hat{\delta}_{\mathrm{mat}}) \\ \mathrm{Cov}(\hat{\delta}_{\mathrm{both}}, \hat{\delta}_{\mathrm{pat}}) & \mathrm{Cov}(\hat{\delta}_{\mathrm{one}}, \hat{\delta}_{\mathrm{pat}}) & \mathrm{Var}(\hat{\delta}_{\mathrm{pat}}) & \mathrm{Cov}(\hat{\delta}_{\mathrm{pat}}, \hat{\delta}_{\mathrm{mat}}) \\ \mathrm{Cov}(\hat{\delta}_{\mathrm{both}}, \hat{\delta}_{\mathrm{mat}}) & \mathrm{Cov}(\hat{\delta}_{\mathrm{one}}, \hat{\delta}_{\mathrm{mat}}) & \mathrm{Cov}(\hat{\delta}_{\mathrm{pat}}, \hat{\delta}_{\mathrm{mat}}) & \mathrm{Var}(\hat{\delta}_{\mathrm{mat}}) \end{bmatrix}.$$

While the diagonal entries are the effect estimate variances for all four groups, the off-diagonal terms depend on the relatedness between each pair of groups. For example, for 'both NT' and 'one NT', we have

$$\mathrm{Cov}(\hat{\delta}_{\mathrm{both}}, \hat{\delta}_{\mathrm{one}}) = (\hat{\mathbf{X}}_{\mathrm{both}}^{\top}\mathbf{V}_{\mathrm{both}}^{-1}\hat{\mathbf{X}}_{\mathrm{both}})^{-1}\hat{\mathbf{X}}_{\mathrm{both}}^{\top}\mathbf{V}_{\mathrm{both}}^{-1}\mathbf{V}_{\mathrm{both:one}}\mathbf{V}_{\mathrm{both}}^{-1}\hat{\mathbf{X}}_{\mathrm{both}}(\hat{\mathbf{X}}_{\mathrm{one}}^{\top}\mathbf{V}_{\mathrm{one}}^{-1}\hat{\mathbf{X}}_{\mathrm{one}})^{-1},$$

where $\mathbf{V}_{\mathrm{both:one}}$ is the block of $\mathbf{V}$ matrix, capturing relatedness between each pair on individual in 'both NT' and 'one NT'.

2.5 The sib-difference estimator

We give details of the sib-difference estimator implemented in *snipar*. Model 3 in the main text only considers two sibs in a family. To account for information from more than two sibs, one common sibship regression model is given by [6]:

$$y_{ik} = \delta(g_{ik} - g_{\mathrm{s}(i)}) + \gamma g_{\mathrm{s}(i)} + \epsilon_{ik}, \tag{7}$$

where g_{ik} and y_{ik} are the gentoypes and phenotype of the kth sib in the ith family; $g_{\mathrm{s}(i)} = \frac{1}{n_i}\sum_{j=1}^{n_i} g_{ij}$ is the average of the genotypes of all n_i sibs in the ith family; γ is the family fixed effect capturing the average genetic effect contributed from all sibs. A similar model that we implemented in *snipar* is:

$$y_{ik} = \delta' g_{ik} + \gamma' g_{\mathrm{s}(i)} + \epsilon_{ik}. \tag{8}$$

We show that (8) is equivalent to (7), i.e., $\delta = \delta'$. To proceed, we first assume there are m families, each with n sibs, and that the true generating model is (7). Let $\mathbf{X}$ and $\hat{\mathbf{X}}$ be such that $[\mathbf{X}]_{i:} = \begin{bmatrix} g_i - g_{\mathrm{s}} & g_{\mathrm{s}} \end{bmatrix}$ and $[\hat{\mathbf{X}}]_{i:} = \begin{bmatrix} g_i & g_{\mathrm{s}} \end{bmatrix}$. Observe that

$$\begin{aligned} \mathrm{Var}(g_i) &= 2f(1-f) \\ \mathrm{Var}(g_{\mathrm{s}}) &= f(1-f)\frac{n + \binom{n}{2}}{n^2} \\ \mathrm{Cov}(g_i, g_{\mathrm{s}}) &= f(1-f)\frac{n+1}{n}. \end{aligned}$$

$$\begin{aligned} \operatorname*{plim}_{m\to\infty} \begin{bmatrix} \hat{\delta}' \\ \hat{\gamma}' \end{bmatrix} &= \operatorname*{plim}_{n_{\mathrm{both}}\to\infty} (\hat{\mathbf{X}}^\top \hat{\mathbf{X}})^{-1} \mathbf{X}^\top \mathbf{y} \\ &= \operatorname*{plim}_{m\to\infty} (\hat{\mathbf{X}}_{\mathrm{mat}}^\top \hat{\mathbf{X}}_{\mathrm{mat}})^{-1} \mathbf{X}_{\mathrm{mat}}^\top [\mathbf{X}_{\mathrm{mat}}]_{i:} \begin{bmatrix} \delta & \gamma \end{bmatrix}^\top \\ &= \mathrm{Var}([\hat{\mathbf{X}}]_{i:})^{-1} \mathrm{Cov}([\hat{\mathbf{X}}]_{i:}, [\mathbf{X}]_{i:}) \begin{bmatrix} \delta & \gamma \end{bmatrix}^\top \\ &= \begin{bmatrix} \mathrm{Var}(g_i) & \mathrm{Cov}(g_i, g_{\mathrm{s}}) \\ \mathrm{Cov}(g_i, g_{\mathrm{s}}) & \mathrm{Var}(g_{\mathrm{s}}) \end{bmatrix}^{-1} \\ &\qquad \cdot \begin{bmatrix} \mathrm{Var}(g_i) - \mathrm{Cov}(g_i, g_{\mathrm{s}}) & \mathrm{Cov}(g_i, g_{\mathrm{s}}) \\ \mathrm{Cov}(g_i, g_{\mathrm{s}}) - \mathrm{Var}(g_{\mathrm{s}}) & \mathrm{Var}(g_{\mathrm{s}}) \end{bmatrix} \begin{bmatrix} \delta \\ \gamma \end{bmatrix} \\ &= \begin{bmatrix} \delta \\ \frac{2n}{n+1}\gamma \end{bmatrix}, \end{aligned}$$

which proves our claim.

2.5.1 Equivalence of the sib-difference estimator to the robust estimator in sibling pair samples

In the main text, we assert that when analyzing samples composed exclusively of sibling pairs without observed parental genotypes, the robust estimator simplifies to the sib-difference estimator. To demonstrate this, note that such samples only consist of the 'one NT' and 'both NT' groups. The sib-regression is applied to the 'one NT' group; thus, it remains to be shown that, for the 'both NT' group, family-based regression yields the same estimates for direct effects as sib-regression.

For sibling pairs in the 'both NT' group, they must be in IBD state 0, so the sum of the siblings' genotypes is equal to the sum of the parental genotypes [1]. Therefore, the sib-regression models for such a sibling pair are

$$y_{i1} = \delta g_{i1} + \gamma g_{s(i)} + \epsilon_{i1} = \delta g_{i1} + \frac{1}{2}\gamma(g_{i1} + g_{i2}) + \epsilon_{i1},$$

$$y_{i2} = \delta g_{i2} + \gamma g_{s(i)} + \epsilon_{i2} = \delta g_{i2} + \frac{1}{2}\gamma(g_{i1} + g_{i2}) + \epsilon_{i2}.$$

In contrast, the family-based regression models are:

$$y_{i1} = \delta g_{i1} + \alpha g_{\mathrm{par}(i)} + \epsilon_{i1} = \delta g_{i1} + \alpha(g_{i1} + g_{i2}) + \epsilon_{i1},$$

$$y_{i2} = \delta g_{i2} + \alpha g_{\mathrm{par}(i)} + \epsilon_{i2} = \delta g_{i2} + \alpha(g_{i1} + g_{i2}) + \epsilon_{i2}.$$

Since the second covariate in the sib-regression models is simply half that in the family-based models, the estimates for direct effects from both approaches are equivalent.

3 Linear imputation using distant relationships

The linear imputation in Equation 1 in the main text used for singletons, ignores information from higher-degree relatives, such as aunts/uncles and cousins. Furthermore, if a dataset contains only singletons with parental genotypes imputed linearly, family-based GWAS will not be possible because the regression design matrix is perfectly collinear. We therefore sought to investigate whether using genotypes of higher-degree relatives could improve imputation of parental genotypes and therefore estimation of direct genetic effects.

In animal breeding, much attention has been given to the problem of estimating breeding values (equivalent to polygenic indices/scores) of un-genotyped members of a pedigree[7–11]. Although these methods have been developed for imputing breeding values, we investigated whether they could be adapted to impute individual SNPs of missing parents by using genotypes of higher-order relatives.

We describe here a method for combining SNP genotype data and a pedigree to impute breeding values[9]: breeding values of animals in the sample are modeled using a multivariate Gaussian distribution, where the covariance matrix is specified by the full numerator relationship matrix (pedigree matrix) with the block for genotyped animals replaced by the corresponding SNP-based GRM; then the breeding values of the ungenotyped animals are estimated using the mean vector conditioned on the breeding values of the genotyped animals. It has been argued that the inclusion of the SNP-based GRM improves prediction because this accounts for Mendelian sampling and better estimates distant relationships.

Similar to their ideas, for each SNP we model all individuals' genotypes and their parents' genotypes as a multivariate Gaussian distribution. (Clearly, SNP genotypes do not have Gaussian distribution, but we only make this assumption in order to derive a computationally tractable conditional expectation formula.) We assume the sparse GRM is readily available to specify covariance among genotyped individuals. But contrary to the animal breeding scenario, full pedigree information is seldom accessible in human biobanks. Therefore, we follow results in [12] and combine information from both the GRM and known parent-offpring relations to specify relatedness coefficients between ungenotyped parents and genotyped individuals.

To evaluate the unbiasedness of the proposed conditional Gaussian imputation and its accuracy relative to the original linear imputation, we performed simulations comparing the imputed values to the true parental genotypes. We consider imputing the parental genotypes of cousin pairs, where the cousin of one individual helps to impute the parents of the other. We followed Young et al.[1] and regressed the true parental genotypes onto the imputed values, with regression coefficients close to 1 if the imputation is unbiased. We also calculated the Pearson correlation coefficients between the true and imputed genotypes, with higher values signifying higher imputation accuracy. Results in Table SN3a show that both the conditional Gaussian method and original linear imputation method are unbiased, and the conditional Gaussian method has slightly higher accuracy. In addition to simulation studies, we compared the performance of the two imputation methods in 894 UKB 'white British' individuals with both parents genotyped. Results in Table SN4 are consistent with simulations.

The proposed conditional Gaussian imputation is more powerful than the naive linear imputation in terms of direct effect estimation – the gain in effective sample size by considering genetic relatedness for singletons can in theory go to infinity because using more distant relatives can prevent collinearity and thereby enable estimation of direct effects. However, the theory derived in Young et al.[1] suggests that direct effect estimates will be biased unless the only indirect genetic effect contribution is from parents. (More generally, the theory suggests that estimates will be biased if the cousin genotype is predictive of the proband phenotype after accounting for proband and parental genotypes.) Again consider the scenario where we impute the parental genotypes of a cousin pair. With a similar simulation setup as in the previous paragraph, we generate phenotypes based on 3 linear models, where the first one involves parental indirect genetic effects and the remaining two also contain indirect genetic effects from cousin and grandparents, respectively. Regression results in Table SN3b are consistent with our expectations from theory. One explanation of this phenomenon is that unlike in the linear imputation scheme, when the parental genotype is imputed using the cousin's genotypes, this results in a higher correlation between the imputed parental genotypes and the cousin's genotypes than that between the true parental genotypes and the cousin's genotypes. This is an issue when the cousin's genotype carries additional information on the offspring's phenotype beyond that contained in the true parental genotype. This is true in the case of indirect genetic effects from grandparents, as the cousin genotype contains information on the grandparents' genotypes that is not contained in parents' genotypes.

It is likely that genotypes of higher degree relatives, such as cousins, in general contain information on the offspring phenotype beyond that contained in the true parental genotype as the result of non-random mating. To investigate this, for different levels of population structure, we simulated cousin pairs in 2 subpopulations and performed parental genotype imputation from cousins using the conditional Gaussian method (Table SN3c and Supplementary Figure 5). Unlike imputation from siblings, imputation from cousins introduced substantial confounding into direct genetic effect estimates even for $F_{st} = 10^{-3}$, resulting in a mean Z^2 statistic of 4.47. The difference with the imputation from siblings is that the sibling genotypes are randomly assigned given the parents, so only contain information on the proband phenotype beyond that in the parental genotypes when there are indirect genetic effects between siblings.

Table SN3: Linear imputation results in simulation.

Imputation	mean regression coefficient (2.5%, 97.5%)	mean correlation coefficient (2.5%, 97.5%)
Linear imputation	1.0008 (0.9890, 1.0207)	0.7072 (0.6989, 0.7158)
Conditional Gaussian	1.0006 (0.9801, 1.0199)	0.7128 (0.7043, 0.7214)

(a) Unbiasedness and accuracy of the two imputation methods for singletons' parents.

Model	mean $\hat{\delta}$ $(-1.96 \cdot s/\sqrt{300}, +1.96 \cdot s/\sqrt{300})$
$y \sim g + 0.5 \cdot g_{\text{par}}$	1.0011 (0.9882, 1.0139)
$y \sim g + 0.5 \cdot g_{\text{par}} + 0.25 \cdot g_{\text{cousin}}$	−0.9347 (−0.9492, −0.9202)
$y \sim g + 0.5 \cdot g_{\text{par}} + 0.25 \cdot g_{\text{grandpar}}$	0.5015 (0.4870, 0.5159)

(b) Direct effect estimates $\hat{\delta}$ obtained from regression onto proband and parental genotypes imputed using the conditional Gaussian method.

F_{st}	mean Z_{δ}^2 $(-1.96 \cdot \hat{\sigma}_{\text{jackknife}}, +1.96 \cdot \hat{\sigma}_{\text{jackknife}})$
0.0	1.0148 (0.9621, 1.0676)
0.001	4.4718 (4.2432, 4.7005)
0.01	32.8545 (31.2964, 34.4126)
0.1	177.0652 (169.6567, 184.4738)

(c) Mean Z_{δ}^2 statistic for direct effect estimates obtained using the parental genotypes imputed using the conditional Gaussian method under different levels of population structure.

Note. (a) We generate 5,000 unrelated cousin pairs with 1,500 independent SNPs; the mean regression coefficient and correlation coefficient are calculated over 1,500 SNPs, with the 2.5% and 97.5% percentiles in the parentheses. (b) For each phenotype-generating model, we simulate 300 datasets with 5,000 unrelated cousin pairs and 1 SNP; $\hat{\delta}$'s are obtained via family-based linear mixed model inference with the expected relatedness coefficients (1/8 for cousin pairs) in the GRM; the empirical mean and confidence interval calculated using the empirical standard error is reported. (c) For each F_{st}, we simulate 2 subpopulations, each with 2,500 unrelated cousin pairs and 3,000 SNPs, where the subpopulation allele frequencies were drawn from the Balding-Nichols model[13]: Beta$(\frac{1-F_{\text{st}}}{2F_{\text{st}}}, \frac{1-F_{\text{st}}}{2F_{\text{st}}})$; imputation of parental genotypes are carried out using the conditional Gaussian method, and non-sampling variance and the corresponding jackknifed confidence interval over 3,000 SNPs are obtained for the direct effect estimates.

Table SN4: Linear imputation results in UKB.

Imputation	mean regression coefficient (2.5%, 97.5%)	mean correlation coefficient (2.5%, 97.5%)
Linear imputation	0.9988 (0.9315, 1.0676)	0.7059 (0.6646, 0.7454)
Conditional Gaussian	0.9968 (0.9295, 1.0649)	0.7178 (0.6768, 0.7567)

Note. Unbiasedness and accuracy of the two imputation methods in UKB. Parental genotype imputation of 894 UKB 'white British' individuals with observed parental genotypes was performed on 27,414 SNPs on chromosome 1. SNPs with minor allele frequency less than 0.05 were removed.

4 Educational Achievement outcome in MCS

MCS contains detailed information on school grades up to age 17. We have produced a measure of educational attainment using the following procedure:

- Calculated the expected exam taking year for each student based on birth year and month.
- For each of GCSE and iGCSE collect the grades achieved for English Language and Mathematics for each student on one row, as well as boolean variables denoting whether they studied for each qualification.
- If a student has an iGCSE but not a GCSE in a subject, use the iGCSE grade to fill in the GCSE grade for that subject. From now on, iGCSEs will be treated like GCSEs.
- Use the population grade distributions for each subject, year and qualification to convert the grades to expected z-scores, where z is a latent variable representing some unobserved, normally distributed notion of educational attainment. To

go from the population grade distribution to expected z-score for each grade, we simulate z from a standard normal distribution, and calculate the mean z for those who would have attained a certain grade. Then, we convert from grade to expected z-score for each subject.

  – If it is not possible to match a student to an appropriate grade distribution, then use the most appropriate other distribution. For example, in the case of iGCSEs, we use the most recent (2016) GCSE grade distribution to avoid bias due to the differing quality of students taking iGCSEs. If a student reports legacy grades for GCSEs when they should report reformed grades, take the most recent (2016) legacy GCSE grade distribution.

- If a student has taken a qualification but has no grade, this will be taken as an indication that the (compulsory) course was not completed, and the student will be excluded from the analysis.
- Let EA equal the mean of the two z-scores.

References

1. Young, A. I. *et al.* Mendelian imputation of parental genotypes improves estimates of direct genetic effects. en. *Nat. Genet.* **54,** 897–905 (June 2022).
2. Cavalli-Sforza, L. L. & Feldman, M. W. Cultural versus biological inheritance: phenotypic transmission from parents to children. (A theory of the effect of parental phenotypes on children's phenotypes). en. *Am. J. Hum. Genet.* **25,** 618–637 (Nov. 1973).
3. Tian, C. *et al.* European population genetic substructure: further definition of ancestry informative markers for distinguishing among diverse European ethnic groups. en. *Mol. Med.* **15,** 371–383 (Nov. 2009).
4. Pritchard, J. K., Stephens, M. & Donnelly, P. Inference of population structure using multilocus genotype data. en. *Genetics* **155,** 945–959 (June 2000).
5. Okbay, A. *et al.* Polygenic prediction of educational attainment within and between families from genome-wide association analyses in 3 million individuals. *Nature Genetics* (2022).
6. Howe, L. J. *et al.* Within-sibship genome-wide association analyses decrease bias in estimates of direct genetic effects. *Nature genetics* **54,** 581–592 (2022).
7. Meuwissen, T. H. E., Luan, T. & Woolliams, J. A. The unified approach to the use of genomic and pedigree information in genomic evaluations revisited. en. *J. Anim. Breed. Genet.* **128,** 429–439 (Dec. 2011).
8. Misztal, I., Legarra, A. & Aguilar, I. Computing procedures for genetic evaluation including phenotypic, full pedigree, and genomic information. en. *J. Dairy Sci.* **92,** 4648–4655 (Sept. 2009).
9. Legarra, A., Aguilar, I. & Misztal, I. A relationship matrix including full pedigree and genomic information. en. *J. Dairy Sci.* **92,** 4656–4663 (Sept. 2009).
10. Aguilar, I. *et al.* Hot topic: a unified approach to utilize phenotypic, full pedigree, and genomic information for genetic evaluation of Holstein final score. en. *J. Dairy Sci.* **93,** 743–752 (Feb. 2010).
11. Christensen, O. F. & Lund, M. S. Genomic prediction when some animals are not genotyped. en. *Genet. Sel. Evol.* **42,** 2 (Jan. 2010).
12. Young, A. I. *et al.* Relatedness disequilibrium regression estimates heritability without environmental bias. *Nature Genetics* **50.** ISSN: 1061-4036. http://www.nature.com/articles/s41588-018-0178-9 (2018).
13. Balding, D. J. & Nichols, R. A. A method for quantifying differentiation between populations at multi-allelic loci and its implications for investigating identity and paternity. en. *Genetica* **96,** 3–12 (1995).
