# Supplementary material for: Family-based genome-wide association study designs for increased power and robustness
Source: Nat Genet. 2025 Mar 10;57(4):1044–52. doi: 10.1038/s41588-025-02118-0 (PMC11985344; doi:10.1038/s41588-025-02118-0)
Supplement: Supplementary file 2 — Reporting Summary [file 41588_2025_2118_MOESM2_ESM.pdf]

nature portfolio

Corresponding author(s): Alexander Strudwick Young, Junming Guan

Last updated by author(s): Dec 4, 2024

Reporting Summary

Nature Portfolio wishes to improve the reproducibility of the work that we publish. This form provides structure for consistency and transparency in reporting. For further information on Nature Portfolio policies, see our Editorial Policies and the Editorial Policy Checklist.

Statistics

For all statistical analyses, confirm that the following items are present in the figure legend, table legend, main text, or Methods section.

n/a	Confirmed	
☐	☒	The exact sample size (*n*) for each experimental group/condition, given as a discrete number and unit of measurement
☒	☐	A statement on whether measurements were taken from distinct samples or whether the same sample was measured repeatedly
☐	☒	The statistical test(s) used AND whether they are one- or two-sided<br>*Only common tests should be described solely by name; describe more complex techniques in the Methods section.*
☐	☒	A description of all covariates tested
☐	☒	A description of any assumptions or corrections, such as tests of normality and adjustment for multiple comparisons
☐	☒	A full description of the statistical parameters including central tendency (e.g. means) or other basic estimates (e.g. regression coefficient) AND variation (e.g. standard deviation) or associated estimates of uncertainty (e.g. confidence intervals)
☐	☒	For null hypothesis testing, the test statistic (e.g. *F*, *t*, *r*) with confidence intervals, effect sizes, degrees of freedom and *P* value noted<br>*Give P values as exact values whenever suitable.*
☒	☐	For Bayesian analysis, information on the choice of priors and Markov chain Monte Carlo settings
☒	☐	For hierarchical and complex designs, identification of the appropriate level for tests and full reporting of outcomes
☐	☒	Estimates of effect sizes (e.g. Cohen's *d*, Pearson's *r*), indicating how they were calculated

Our web collection on statistics for biologists contains articles on many of the points above.

Software and code

Policy information about availability of computer code

Data collection	We only used publicly available datasets and therefore did not conduct data collection.
Data analysis	For the UK Biobank GWAS analysis, we used a development branch of the Python package snipar to perform IBD state inference, parental genotype imputation and family-based GWAS (available at https://zenodo.org/records/14270274); we used the sparse LU solver for matrix inversion and the L-BFGS algorithm for variance component estimation, both implemented in the SciPy Python package[v1.7.1]; we used KING[v2.2.5] to perform IBD segment and relationship inference; we computed genetic correlations between direct genetic effect estimates from our prososed methods for height and educational attainment respectively using LDSC[v1.0.1].<br>For polygenic prediction in the Millennium Cohort Study: we used the OADP algorithm implemented in the R package bigsnpr[v1.12.2] (https://privefl.github.io/bigsnpr/) to infer sample ancestries; we used PRSCS[Jun 4, 2021] to calculate SNP weights; we used the pgs.py script in snipar to estimate direct effects of the PGIs.

For manuscripts utilizing custom algorithms or software that are central to the research but not yet described in published literature, software must be made available to editors and reviewers. We strongly encourage code deposition in a community repository (e.g. GitHub). See the Nature Portfolio guidelines for submitting code & software for further information.

Data

Policy information about availability of data

All manuscripts must include a data availability statement. This statement should provide the following information, where applicable:

- Accession codes, unique identifiers, or web links for publicly available datasets
- A description of any restrictions on data availability
- For clinical datasets or third party data, please ensure that the statement adheres to our policy

Summary statistics from the different estimators applied to UK Biobank data are available for download from the SSGAC data portal: https://thessgac.com/. Applications for access to the UKB data can be made on the UKB website (http://www.ukbiobank.ac.uk/register-apply/). Applications for Millennium Cohort Study data can be made by following the instructions here: https://cls.ucl.ac.uk/data-access-training/data-access/accessing-data-directly-from-cls/. 1000 Genomes phase 3 data can be downloaded using the download_1000G function provided by the bigsnpr R package.

Research involving human participants, their data, or biological material

Policy information about studies with human participants or human data. See also policy information about sex, gender (identity/presentation), and sexual orientation and race, ethnicity and racism.

Reporting on sex and gender	Results in this study apply to both sexes except for sex-specific phenotypes. Sex was determined based on self-reporting and genotype data as described in the documentation on the UK Biobank and MCS datasets.
Reporting on race, ethnicity, or other socially relevant groupings	For the UK Biobank data, we classified individuals into the 'white British' ancestry subsample using the flag provided by UK Biobank. For the Young et al. and unified estimator, we used individuals classified as 'White British' to make our results comparable to existing genome-wide association studies and to control for population structure.<br><br>For the the Millennium Cohort Study, we classified individuals into European (EUR) and South Asian (SAS) using the OADP and KNN algorithms. We projected individuals onto genetic principal components derived from 1000 Genomes data and classified individuals as belonging to the EUR or SAS superpopulations if all 20 of their nearest neighbors in the 1000 Genomes data were from the EUR or SAS samples. We referred to these samples as 'EUR' and 'SAS' samples.
Population characteristics	The UK Biobank project is a prospective cohort study with deep genetic and phenotypic data collected on approximately 500,000 individuals from across the United Kingdom, aged between 40 and 69 at recruitment. The Millennium Cohort Study is a representative sample of individuals born in the UK in the year 2000 and their parents.
Recruitment	Recruitment is not applicable since we used existing datasets.
Ethics oversight	UK Biobank has approval from the North West Multi-centre Research Ethics Committee (MREC) as a Research Tissue Bank (RTB) approval. This approval means that researchers do not require separate ethical clearance and can operate under the RTB approval (there are certain exceptions to this which are set out in the Access Procedures, such as re-contact applications). The Millennium Cohort Study has obtained ethical approval from NHS Research Ethics Committees (RECs).

Note that full information on the approval of the study protocol must also be provided in the manuscript.

Field-specific reporting

Please select the one below that is the best fit for your research. If you are not sure, read the appropriate sections before making your selection.

☒ Life sciences ☐ Behavioural & social sciences ☐ Ecological, evolutionary & environmental sciences

For a reference copy of the document with all sections, see nature.com/documents/nr-reporting-summary-flat.pdf

Life sciences study design

All studies must disclose on these points even when the disclosure is negative.

Sample size	We used genotyped individuals in the UK Biobank passing filters corresponding to specific methods:<br>For the sib-difference method, we used all genotyped individuals passing quality control filters with at least one genotyped sibling: 46,698;<br>For the robust estimator, we used all genotyped individuals passing quality control filters with at least one non-transmitted parental allele observed: 51,875;<br>For the Young et al. estimator, we used all genotyped individuals of 'White British' ancestry (identified by the UK Biobank), passing quality control filters, and with at least one genotyped first-degree relative: 44,570;<br>For the unified estimator, we used all genotyped individuals of 'White British' ancestry (identified by the UK Biobank) and passing quality control filters: 408,254.<br>For the Millennium Cohort Study, we used all genotyped individuals of either European or South Asian ancestry identified by the OADP and KNN algorithms: 10416.
Data exclusions	For analyses on UK Biobank, we filtered out individuals identified by UK Biobank as having excess relatives, excess heterozygosity, sex chromosome aneuploidy, and excess genotype missingness.

For the Millennium Cohort Study, we restricted our sample to individuals of European or South Asian ancestry identified by the OADP and KNN algorithms, to make our analyses comparable to existing studies on cross-ancestry polygenic prediction.

Replication	Our UK Biobank analysis was performed to show the relative power of the different estimators in a large-scale biobank. Direct replication of these results is not relevant to the conclusions of our study. However, our Millennium Cohort Study analysis demonstrates that our UK Biobank analysis produced results with external validity by demonstrated statistically significant out-of-sample prediction ability for genetic predictors derived from our UK Biobank analysis.
Randomization	Genetic materials are randomized during meioses; this randomization is used in family-based GWAS designs to remove confounding.
Blinding	Blinding is not applicable since we did not compare experimental groups.

Reporting for specific materials, systems and methods

We require information from authors about some types of materials, experimental systems and methods used in many studies. Here, indicate whether each material, system or method listed is relevant to your study. If you are not sure if a list item applies to your research, read the appropriate section before selecting a response.

Materials & experimental systems

n/a	Involved in the study
☒	☐ Antibodies
☒	☐ Eukaryotic cell lines
☒	☐ Palaeontology and archaeology
☒	☐ Animals and other organisms
☒	☐ Clinical data
☒	☐ Dual use research of concern
☒	☐ Plants

Methods

n/a	Involved in the study
☒	☐ ChIP-seq
☒	☐ Flow cytometry
☒	☐ MRI-based neuroimaging
